# Deep Neural Decision Forest (DNDF): A Novel Approach for Enhancing Intrusion Detection Systems in Network Traffic Analysis

**DOI:** 10.3390/s23208362

**Published:** 2023-10-10

**Authors:** Fatma S. Alrayes, Mohammed Zakariah, Maha Driss, Wadii Boulila

**Affiliations:** 1 Information Systems Department, College of Computer and Information Sciences, Princess Nourah bint Abdulrahman University, Riyadh 11671, Saudi Arabia; fsalrayes@pnu.edu.sa; 2 College of Computer and Information Sciences, King Saud University, Riyadh 11362, Saudi Arabia; mzakariah@ksu.edu.sa; 3 Robotics and Internet-of-Things Laboratory, Prince Sultan University, Riyadh 12435, Saudi Arabia; wboulila@psu.edu.sa; 4 RIADI Laboratory, National School of Computer Sciences, University of Manouba, Manouba 2010, Tunisia

**Keywords:** network traffic analysis, deep neural decision forest (DNDF), CICIDS 2017 dataset, deep learning, network security, machine learning

## Abstract

Intrusion detection systems, also known as IDSs, are widely regarded as one of the most essential components of an organization’s network security. This is because IDSs serve as the organization’s first line of defense against several cyberattacks and are accountable for accurately detecting any possible network intrusions. Several implementations of IDSs accomplish the detection of potential threats throughout flow-based network traffic analysis. Traditional IDSs frequently struggle to provide accurate real-time intrusion detection while keeping up with the changing landscape of threat. Innovative methods used to improve IDSs’ performance in network traffic analysis are urgently needed to overcome these drawbacks. In this study, we introduced a model called a deep neural decision forest (DNDF), which allows the enhancement of classification trees with the power of deep networks to learn data representations. We essentially utilized the CICIDS 2017 dataset for network traffic analysis and extended our experiments to evaluate the DNDF model’s performance on two additional datasets: CICIDS 2018 and a custom network traffic dataset. Our findings showed that DNDF, a combination of deep neural networks and decision forests, outperformed reference approaches with a remarkable precision of 99.96% by using the CICIDS 2017 dataset while creating latent representations in deep layers. This success can be attributed to improved feature representation, model optimization, and resilience to noisy and unbalanced input data, emphasizing DNDF’s capabilities in intrusion detection and network security solutions.

## 1. Introduction

The goal of the subset of traffic analysis methodologies, known as traffic classification, is to categorize traffic flow into several predetermined groups, such as normal or abnormal traffic and the application type [1]. It makes it easier for Internet service providers to manage their infrastructures effectively and meets the requirements for quality of service [2,3,4]. The first traffic processors used each application’s port number [3] to determine who it was. By examining the packet header, this approach exclusively reveals the port numbers and their correspondences. Due to this, the method that looks at port numbers turned out to be the fastest and easiest [4]. However, there are some problems with this plan. Some programs can hide from network security measures by using dynamic port numbers or ports tied to multiple protocols. This makes port-based algorithms less accurate [5,6].

In the current digital era, the increasing complexity and frequency of cyber threats—which include network breaches, data theft, and cyberattacks—pose serious risks to people, businesses, and entire countries. To combat these threats, there is an urgent need for IDSs that are more effective. Traditional IDS techniques struggle to keep up with cyber attackers’ constant innovation of evasion strategies. A promising method for identifying complex and evolving attack patterns that frequently escape conventional systems is provided by deep learning models.

Deep packet inspection (DPI) methods [7,8,9,10] have been developed to enable a deeper understanding of port numbers, moving beyond their face value interpretation. It looks at all the packet data, which uses a lot of central processing unit (CPU) resources, can be hard to scale, and needs protected data to be correctly identified. Machine learning (ML) lets us model, learn, and find complex and hidden trends in network traffic behavior using training data [11]. It can help solve the problems that have been identified. Simple and ensemble models are the two main categories that can broadly classify ML-based models [10,11,12,13]. Ensemble models seek to integrate heterogeneous or homogeneous (often, classifiers) to produce a model that outperforms each of the individual models and overcomes the limits of each individual model [14,15,16]. More precisely, several alternative homogeneous ensemble frameworks, such as bagging (e.g., random forest (RF)) and boosting (e.g., XGBoost), have been presented, with the majority of them relying on the decision tree (DT) model [8,15,17].

On the other hand, a heterogeneous ensemble has been proposed to use the models’ varied benefits. Blending and majority voting are two instances of distinct models [18]. The blending process is divided into two stages: the underlying classifier and the meta-classifier. Blending is a powerful ensemble approach because it combines the base classifier with a meta-classifier, which is then merged with the base classifier [19]. On the other hand, the application of the blended ensemble for network traffic categorization has received comparatively little attention [20]. Deep learning (DL) is also well recognized for outperforming standard shallow ML models in a range of sectors, including healthcare, computer vision, and network resource management. DL has also had success with network traffic categorization. DL is a subset of ML that evolved from neural networks (NNs), and it has a unique nature and set of characteristics for handling difficult tasks. Furthermore, the DT algorithm is well known for its simplicity, making it straightforward for human professionals to grasp. It is one of the best learning algorithms for classifying network traffic [20,21].

This paper focuses on a unique approach for accurate network traffic analysis by utilizing the CICIDS 2017 dataset [2,7,8,9]. We justify our choice to do so by citing the reasons described in [12,17,22]. Several research projects have focused on developing intrusion, botnet, and virus detection systems based on deep learning networks [23]. The original artificial neural network (ANN) design serves as the foundation for deep learning networks (DLN). This design includes a multilayer architecture in addition to activation and optimization methods [24].

To build attack detection rules, deep learning-based intrusion detection simply needs a small number of attack signatures or a short list of common actions [25]. By utilizing a quantifiable characteristic of the monitored network traffic feature, the deep learning model is trained with empirical data to identify network assaults. It is accomplished via “empirical data training”. DL models have been gradually used in intrusion detection during the past several years [21] to improve classification classifiers. This can be ascribed to the astounding effectiveness and simplicity of DL models’ use. The class imbalance significantly negatively impacts classification results [26], which happens when real-time network intrusion detection is used. The difference between dominant and weaker classes cannot be distinguished by models that exclusively forecast dominant classes [27]. Class imbalance issues are commonly solved using resampling techniques [28,29]. The use of oversampling techniques is not without drawbacks, though. The original data can be harmed by oversampling [30]. The model may require more time to train when oversampling techniques are used. Inadequate sampling might result in the loss of important data, which can make classification difficult [31,32].

**Figure 1:**
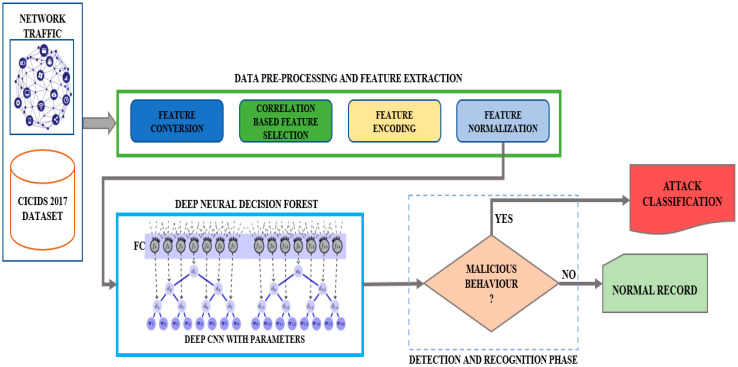
Framework for DNDF model using CICIDS 2017 dataset for network analysis.

The network analysis framework for the DNDF model, which essentially uses the CICIDS 2017 dataset, is shown in Figure 1. We extended our experiments to evaluate the DNDF model’s performance on two additional datasets: CICIDS 2018 and a custom network traffic dataset. This study aimed to construct a robust network intrusion detection system through experimentation with diverse datasets. Using a multi-categorization approach, the system relies on flow-based statistics to detect and classify various attack types. To identify the type of network traffic, we developed a deep neural decision forest network model. The main contributions that this paper makes are listed below.

- DNDF Architecture: DNDF maximizes the advantages of both decision forest and deep neural network models. Combining deep learning techniques with decision forest models’ interpretability and ensemble capabilities enables the extraction of complicated attributes and patterns from network traffic data.
- Enhanced Feature Representation: DNDF uses cutting-edge feature representation algorithms to capture the complex properties of network traffic data accurately. Classification accuracy is improved with DNDF by extracting and encoding high-level data that permits more effective separation between different network activities.
- Model Optimization: The DNDF uses cutting-edge optimization techniques developed especially for deep learning models and decision forests. By assisting in optimizing the decision limits and fine-tuning the model’s parameters, these strategies lead to more accurate predictions and less overfitting.
- Robustness to Noisy and Unbalanced Data: DNDF is built to handle noisy and unbalanced network traffic datasets effectively. It employs techniques such as data augmentation, oversampling, and undersampling to balance out the distribution of classes and increase the model’s tolerance to noise and outliers in the data.
- Evaluation through Diverse Datasets: Our proposed network analysis framework using the DNDF model was primarily based on the CICIDS 2017 dataset. Our research expanded into multiple experiments assessing the DNDF model’s performance, including evaluations on two supplementary datasets: CICIDS 2018 and a custom network traffic dataset. We aim to develop a robust network intrusion detection system by comprehensively exploring various datasets.

Our proposed DNDF method surpassed all reference methods on the CICIDS 2017 dataset with an accuracy of 99.96%. This remarkable improvement may be caused by several factors, as stated above.

The following is the structural scheme for the remaining parts of this work: In Section 2, we will go through related works. In Section 3, we will present the used dataset and the performed preparation operations. In Section 4, we will talk about the proposed approach. The experimental details and discussion are presented in Section 5 and Section 6, followed by a comparison. Lastly, the summary and future research directions are presented in Section 7.

## 2. Literature Review

Several other scholars have employed ML in ways comparable to our presented method to evaluate network traffic. In this part of the article, we will examine and compare their methods to ours, pointing out the key differences. In the first part of this presentation, we will talk about researchers who have classified benign traffic data for network quality assurance using data preparation approaches comparable to ours.

ML analysis was suggested by [7] as a way to enhance network security while lowering the expense associated with preprocessing. In this instance, the raw network data was first converted into bitmap files to find possibly malicious behavior and then it was processed by a two-dimensional convolutional neural network (2D-CNN) model. Based on tests with three publicly available network traffic datasets, their model successfully identifies various malicious traffic patterns, including zero-day attacks. The researchers looked at how well their model worked before coming to this conclusion. This technology is highly suited for doing on-the-fly network traffic analysis to identify malicious traffic flows since the cost of preprocessing the network data to use the 2D-CNN model is incredibly inexpensive. On the CICIDS 2017 dataset, the first reference method, 2D-CNN, obtained an accuracy of 90.6%. This approach, which seeks to achieve this goal, aims to identify malicious network traffic with the least amount of preprocessing expense.

In addition, ref. [8] introduced D.S. sampling, a fresh method for balancing datasets. This strategy was inspired by the Synthetic Minority Oversampling Technique (SMOTE) algorithm. Their solution separated the sample into easy- and hard-to-classify subsets, with the latter being the only one to be balanced, in contrast to the method. The concepts of oversampling and undersampling were combined into a cohesive framework, avoiding the overgeneralization that the SMOTE brought. Given that adopting a hierarchical structure may lead to better categorizing the minority of abnormal traffic, a two-layer structure coupled with XGBoost and the random forest has been proposed as an additional technique for the multiclassification of anomalous data. The CICIDS 2017 dataset was used for the tests, and the findings are shown in their article. Based on the findings, it was determined that the model under investigation had a classification accuracy of greater than 99.70%.

Another work [9] also investigated several ML approaches to determine which may deliver the best traffic categorization outcomes based on classification, performance metrics, and execution times. This analysis used the CICIDS 2017 dataset since it provided bidirectional traffic flows that included both traffic that was thought to be innocuous and the traffic that included a variety of current attacks.

In the initial investigation, the authors employed decision-tree-based algorithms, achieving F1 values exceeding 0.999, demonstrating the effectiveness of their proposed approach in integrating raw network traffic samples from the CICIDS 2017 dataset. They discovered that binary classification produced better and quicker results as process complexity reduced when simply taking the most crucial factors into account. With F1 values more than 0.997 and quick execution durations, the classification outcomes employing tree-based approaches were robust. They verified that tree-based ML methods like PART or J48 might be viable substitutes to the RF methodology for flow-based intrusion detection with a 99.86% accuracy on the CICIDS 2017 dataset.

In a different study, the authors suggested utilizing the RF approach for feature selection and figuring out the best threshold to accurately classify DoS assaults with a 99.83% accuracy rate on the CICIDS 2017 dataset.

In a different approach, ref. [22] combined three methodologies to achieve an accuracy range of 99.7%. These methodologies included examining feature correlations, using the T-distributed stochastic adjacent embedding data dimensional reduction method, and applying the R.F. technique to examine the accuracy and false positive rate complexities.

James Adeke and colleagues [17] used ML and the RF method to classify whether the user datagram protocol and transmission control protocol accurately flow in the network, achieving an average performance accuracy of 99.52%.

The Adaptive Convolutional Neural Network Structure for Network Traffic Classification (ACNNS-NTC) was developed by Zhuang Han et al. [21] and showed above 99% accuracy on publicly accessible datasets such as the ISCX-IDS2012, USTC-TFC2016, and CIC-IDS2017.

An SDN-based modular architecture was created in another research [33] to identify Distributed Denial-of-Service (DDoS) attacks at the transport and application levels. On the CIC DoS 2017 and CICD DoS 2019 datasets, they classified unknown traffic with up to 99% accuracy using multiple ML and DL approaches.

Choobdar et al. achieved 98.5% average accuracy in attack detection and classification using SDNs and a three-stage procedure on NSL-KDD and CICIDS 2017 datasets [34].

Convolutional neural networks and gated recurrent units were coupled by Henry et al. [35] to optimize network parameters, resulting in 98.73% accuracy and a 0.075 false positive rate (FPR) on the CICIDS-2017 dataset.

Finally, intrusion detection using raw network traffic in computationally constrained contexts was performed using transfer learning with neural networks. By using only 5000 training samples on edge devices, the combination of a transferred one-dimensional convolutional neural network model and a retrained random forest model was able to outperform with a 96% accuracy on the UNSW-NB15 and CICIDS 2017 datasets.

The following sections are the main contributions of highlighted studies.

This research proposes increment, a measure of long short-term memory (LSTM) that is derived as the function and derivative product. Additionally, the LSTM, regarded as an incremental LSTM, is subject to state change. Finally, we used trials to examine how the state change affected the performance of the incremental LSTM. The incremental LSTM-based intrusion detection system has greater accuracy than previous methods, according to experiments [2].

This suggested study introduces a novel technique employing an extended deep reinforcement learning (EDRL) algorithm to improve network traffic analysis and prediction. This effort is significant because it will help with intelligence-based network traffic prediction and network management problems. An experiment was conducted to evaluate the EDRL’s accuracy, precision, and false positive and false negative characteristics. Additionally, deep learning algorithms and CNN machines have been utilized to forecast various kinds of network traffic, including unencrypted and encrypted data traffic as well as text- and video-based traffic [4].

This study uses minimal preprocessing overhead ML analysis for network security. Raw network data are instantly transformed into bitmap files to detect fraudulent traffic and are analyzed using a 2D-CNN model. Based on testing with three open-source network traffic datasets, the model accurately detects various malicious traffic flows, even zero-day assaults. The 2D-CNN model is excellent for on-the-fly network traffic analysis for malicious traffic flows since the overhead of preparing the network data before applying it is relatively minimal [7].

Based on the SMOTE algorithm, this research suggests a brand-new dataset-balancing technique called SD sampling. The SMOTE algorithm splits samples into two categories—easily classifiable and difficultly classifiable—and only balances the difficultly classifiable samples. This solution avoids the overgeneralization of the SMOTE algorithm and combines the concepts of oversampling and undersampling. Additionally, employing a hierarchical structure may better classify minority aberrant traffic. A two-layer structure paired with XGBoost and the random forest is presented for the multiclassification of anomalous data. In this stud, experiments are conducted on the CICIDS 2017 dataset [8].

Weka software (4.2.x) has tested several classification methods, including naive Bayes, logistic, multilayer perceptrons, sequential minimum optimization, k-nearest neighbors, adaptive boosting, OneR, J48, PART, and random forest. As a general conclusion, decision-tree-based approaches (PART, J48, and random forest) proved to be the most effective, with F1 values over 0.999 (an average value was derived for the whole dataset). Additionally, binary classification (distinguishing only between normal traffic and attack) and multiclass classification (differentiating between various attack types) were compared, and the outcome of reducing the number of attributes using the correlation-based feature selection (CFS) technique was assessed [9].

This study uses ML and parameter optimization to accurately classify the network’s user datagram protocol (UDP) and transmission control protocol (TCP) flows. The Waikato Environment for Knowledge Analysis (WEKA, 4.2.x) software’s three randomly chosen ML algorithms were used to validate the approach using 10 folds of cross-validation, and the algorithm with the highest performance was picked. Three scenarios were used in experiments utilizing the USTC-TFC2016 dataset [17].

The study of [36] illustrates the viability of transfer learning for intrusion detection in computationally constrained contexts utilizing raw network traffic. Our findings demonstrate this when a retrained random forest model is used in conjunction with a transferred one-dimensional convolutional neural network model [37,38,39,40].

A novel Lightweight Double-Stage Scheme for identifying malicious Domain Name System (DNS) over HTTPS traffic is introduced in a recent study by Abu Al-Haija et al. [41]. The method uses a hybrid learning approach and provides encouraging insights into better detection techniques for secure network communications. A one-class classifier model for memory dump malware detection is presented by Al-Qudah et al. [42] in their research. This model demonstrates how it might improve memory dump analysis methods for malware detection, thus advancing cybersecurity.

The Abu Al-Haija and Al-Badawi [43] study introduced a system for routing attack-aware Internet of Things (IoT) network traffic. This method addressed the difficulties of IoT traffic routing while considering potential threats by utilizing ensemble learning, improving the security of IoT networks. An intrusion detection and classification system with improved data engineering was proposed by Alsulami et al. [44] for IoT traffic. This system, which they discuss in their research, shows enhanced abilities in identifying and categorizing intrusions within IoT networks, enhancing their overall security posture.

Table 1 contains related works describing the proposed methods, the used datasets, and the obtained performance.

Numerous significant research gaps in the areas of network traffic analysis and intrusion detection were found as a result of the thorough literature review that was conducted for this study. ML and DL techniques need to be more accurate and efficient to deal with large datasets and complex traffic patterns. Additionally, the problem of dealing with imbalanced data distributions—where legitimate traffic far outweighs malicious traffic—remains a crucial factor that calls for creative solutions to avoid overgeneralization. A clear understanding of the relative performance of different ML and DL approaches in various scenarios is made more difficult by the lack of thorough comparisons between various ML and DL approaches. The transition from theoretical performance metrics to actual implementation of intrusion detection systems has also received little attention, even though it is crucial to comprehend the implications of their deployment in the real world. Another research challenge is adapting IDSs to detect new and emerging attack patterns, especially zero-day attacks. By introducing a cutting-edge method called DNDF, this study significantly adds to the body of knowledge on IDS and network traffic analysis. IDSs are essential for maintaining organizational network security because they act as the first line of defense against online dangers. However, in the face of evolving cyber threats, traditional IDSs frequently struggle to deliver accurate and real-time intrusion detection. To improve the classification of network traffic data, the DNDF model described in this study combines the advantages of deep neural networks and decision forests. The model’s ability to produce latent representations within deep network layers is demonstrated by the experimentation using the well-known CICIDS 2017 dataset. A detailed description of the proposed model is presented in the subsequent sections.

## 3. Datasets

We primarily employed the CICIDS 2017 dataset for network traffic analysis and expanded our experiments to assess the DNDF model’s performance on two additional datasets: CICIDS 2018 and a custom network traffic dataset.

### 3.1. CICIDS 2017 and 2018

The data used for our study were from the CICIDS 2017 and the CICIDS 2018 datasets, sizable collections of network traffic data made especially for intrusion detection and network traffic analysis operations. These datasets contain numerous network traffic scenarios, such as typical traffic and various assaults and irregularities.

The CICIDS 2017 dataset was gathered in a controlled experimental environment that featured numerous network activities and traffic simulations. It comprises network traffic flows that reflect both good and bad traffic scenarios and are captured in a real-world network environment [45]. The dataset contains a broad range of features extracted from network traffic and is highly beneficial in studying the characteristics and behaviors of different network activities.

The CICIDS 2017 data frame can be seen in Table 2. The network traffic was obtained from some sources, including actual network scenarios, network simulations, and attack simulations, to ensure the dataset’s validity and applicability. The raw network packets were captured during the data gathering process using sensors and network monitoring tools, and they were afterward turned into a structured dataset for additional analysis. The obtained dataset includes elements allowing in-depth analysis and classification of network traffic patterns, such as flow-level properties, statistical measurements, payload characteristics, and packet-level data. This dataset also provides labeled ground truth data demonstrating the existence of different network activities, including ordinary traffic, assaults (including DDoS and port scanning), and anomalies. The CICIDS 2017 dataset’s properties provide useful information for network traffic analysis, as shown in Table 3.

A class label was assigned based on the type of network activity that each instance of the CICIDS 2017 dataset represents. The network traffic categorization algorithms can be trained and evaluated using the class labels, which provide real-world data.

The most recent version of the dataset, CICIDS 2018, has the same feature set as CICIDS 2017 with the exception of one additional feature: timestamp. However, it is enriched and more comprehensive, maintaining the same three-label distribution. This extensive dataset has a sizable size and is described as shown by Table 4.

The CICIDS 2017 and CICIDS 2018 datasets were used in this study to train and assess the deep neural decision forest (DNDF) model for network traffic analysis. Using these datasets, we studied the characteristics and patterns of network traffic flows, identified potential dangers or anomalies, and developed a robust classification model.

By employing the rich characteristics and label information of the CICIDS 2017 and CICIDS 2018 datasets, we conducted an in-depth study of network traffic and accurately characterized various types of network activity. These datasets made a substantial contribution to the development of intrusion detection and network traffic analysis, as well as to the validity and dependability of the findings of our study.

### 3.2. Custom Network Traffic Dataset

Wireshark, a flexible program that can examine traffic from a variety of sources, including Wi-Fi, Bluetooth, and Ethernet connections, was used to create our customized network traffic dataset. In this study, Wi-Fi and Ethernet connections were particularly utilized to assess the traffic of three computer systems under normal conditions.

We instantly collected and recorded traffic data using Wireshark, which helped us produce a comprehensive table with information on protocols, packet durations, payloads, source IP addresses, and destination IP addresses. To simulate malicious network traffic over the transmission network, we used metaplots. The introduced intrusion traffic includes XSS (Cross-Site Scripting) and SQL injection.

Three real-time scenarios were used in the investigation, enabling us to examine both normal and intrusion-simulated transmission traffic.

1. We captured normal network traffic over the Internet via Ethernet and Wi-Fi connections using Wireshark.
2. We utilized Metasploit to simulate XSS (Cross-Site Scripting) network traffic, and Wireshark captured the traffic on the host computer.
3. We simulated SQL injection traffic using Metasploit, with Wireshark capturing the traffic on the host computer.

**Figure 2:**
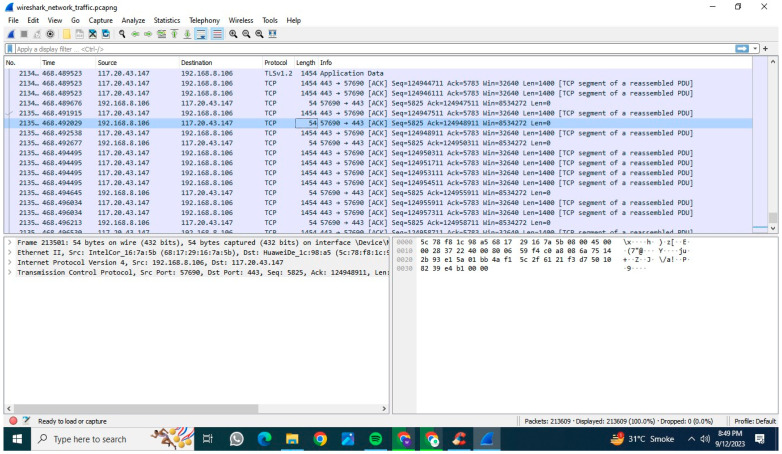
Network traffic generation with Wireshark.

We tested multiple types of traffic in our transmission network tests and examined the results. The transmission dataset was recorded using Wireshark and saved as a pcapng file. Later, this file was used to create models and extract features. Figure 2 shows a screenshot of a network traffic generation with Wireshark.

To make our network traffic dataset more comprehensive, we have expanded its feature set. However, as exposing our PC to real malicious traffic is not advisable, this dataset does not include malware traffic initially. We used Metasploit, a different piece of software, to simulate various types of attacks to address this, and Wireshark was used to analyze the resulting traffic. We specifically utilized XSS (Cross-Site Scripting) and SQL injection attacks. In order to capture normal, XSS, and SQL injection traffic, Wireshark created files in the CSV and pcapng formats. The dataset’s comprehensiveness improved once these output files were placed into a Python environment for additional feature extraction. We retrieved three distinct types of features from this dataset. These types are described in the following list.

- **Packet-level features:**
  ○ Interarrival times: calculate the time interval between consecutive packets.
  ○ Packet size statistics: extract statistics such as mean, median, and standard deviation of packet sizes within a flow.
  ○ Direction of traffic: analyze the direction of network traffic, including statistics such as mean, median, and standard deviation of packet sizes within a flow.
- **Flow features:**
  ○ Number of packets per flow: count the total number of packets in a flow.
  ○ Duration of flows: calculate the time duration of each network flow.
  ○ size of flows: compute statistics (e.g., mean, max, min) related to the size of data transferred in each flow.
- **Protocol features:**
  ○ Protocol counts: create features that count the usage of different protocols in network traffic.
  ○ Statistical relevance to the protocol: Calculate unique statistics for each protocol, such as the quantity of HTTP requests within HTTP flows.

After extracting these features, we concatenated all data frames to create a comprehensive dataset of all labels, i.e., normal, XSS, and SQL injection.

The dataset underwent label encoding, creating two classes (‘normal’ and ‘attack’), encompassing both the SQL injection and XSS. The labeling is also performed by considering the length of payloads because most malicious attacks have a longer length of payload information.

In this custom dataset, there are a total of 15 features along with one target column. The features include ‘Time’, ‘Source’, ‘Destination’, ‘Protocol’, ‘Length’, ‘Info’, ‘Flow_Duration’, ‘Num_Packets_Per_Flow’, ‘Flow_Size’, ‘MinPacketLength’, ‘MaxPacketLength’, ‘AvgPacketLength’, ‘TotalPackets’, and ‘TotalBytes.’ While additional features such as time-based attributes, payload analysis, and aggregated features can be extracted, the existing feature set provides satisfactory performance for the scope of this study.

The final data frame is shown in Table 5.

### 3.3. Data Preprocessing

Data pretreatment is just one of the crucial stages in preparing the dataset for analysis and modeling. For network traffic analysis utilizing the CICIDS 2017 dataset, the following are some typical data preparation techniques.

#### Data Cleaning

In this step, we performed the removal of duplicate records from the dataset. We also addressed incomplete values by either imputing them or removing instances where they existed based on the extent of missing data and its impact on the analysis.

The number of empty elements in our data frame is listed below in Table 6.

The modified data frame after deleting these null elements is shown in Table 7 that follows. These null entries are eliminated using Pandas’ drop method. Currently, none of our data frame’s variables have any null entries.

### 3.4. Feature Selection

In this step, we performed an analysis of each component of the dataset to assess its value and significance. We then selected the informative attributes from this subset, focusing on those crucial for network traffic analysis. Our selection process was guided by techniques such as correlation analysis, feature importance ranking, and domain expertise. Furthermore, we employed automated feature selection methods, such as recursive feature elimination (RFE), selectKBest, and principal component analysis (PCA), to identify the most relevant characteristics.

#### Correlation-Based Feature Selection

We employed a correlation analysis-based technique to locate highly linked features in the dataset during the feature selection stage. While minimizing redundant information, the goal was to select the most informative subset of attributes. The best traits for this study’s feature selection were chosen using the Pearson coefficient approach. A total of 40 characteristics have been selected out of 76. In the following points, we list the steps that made up this procedure:

- Calculate the Correlation Matrix: We initially calculate the dataset’s correlation matrix using the corr() method. The correlation matrix calculates the pairwise correlation between each pair of features in the dataset. The linear correlation between the variables is evaluated numerically.
- Set the Correlation Threshold: We set a threshold value to establish the level at which two attributes are deemed highly connected. The threshold is now set at 0.8, although it might change depending on the demands of this study. The thirty-nine identified correlated features are displayed in Table 8.
- Finding Highly Correlated Features: Using the correlation matrix and the threshold, we compare each correlation value to the threshold to determine the highly correlated features. We use the np where () method to obtain the indices of the objects that satisfy the criterion.
- Obtain Unique Feature Pairs: We extract the unique feature pairs from the highly linked qualities. By iterating through the feature indexes, the feature names for each index of a highly correlated feature are derived from the columns of the data frame. We take care only to consider one pair of qualities for each identical pair, and we make sure that no two separate pairs of features are linked together.
- Create a New Data Frame: We create a new data frame (new_df) by selecting the remaining features from the original data frame after deleting the strongly related features. The set difference between the columns in the original data frame and the features discovered in the feature pairs is used to do this.
- Print the New Data Frame: Finally, we print the new data frame to confirm that the strongly correlated features were removed and the remaining features are appropriately depicted.

By locating and removing strongly correlated features, we are able to eliminate redundancy, enhance the functionality of our model, and make it easier to understand. We can concentrate on the most important and independent factors for our network traffic analysis by choosing a subset of features that are less connected with one another. We used the Pearson coefficient approach to select the most appropriate features for this investigation. Out of 76 features, a total of 40 have been chosen.

### 3.5. Feature Encoding

In this step, we normalized numerical attributes to achieve consistent measurement scales. This was achieved by applying either min-max scaling or standardization techniques, ensuring that feature values align with a specific range, mean, or standard deviation. Specifically, we standardized the selected features in both the training and testing sets using a regular scalar operation. You can find the data frame displaying standardized features in Table 9.

The three classes in the label are as follows:

BENIGN 238221.
PortScan 158804.
DDoS 128025.

The label encoding translates these labels into a numerical type as an array ([0, 0, 1, …, 1, 1, 2]).

#### Data Scaling and Normalization

The aim of this step is to normalize the numerical properties to ensure they are all measured on the same scale. Min-max scaling or standardization is used to scale the feature values to a specific range, mean, or standard deviation. Here, we converted the chosen features in the training and testing sets to standard features using a regular scalar operation. The data frame for standardized features is also shown in Table 10.

Each feature is modified individually with the StandardScaler to have a mean and variance of 0 and 1, respectively. The traits are, therefore, changed to have a mean of 0 and a standard deviation of 1. This must be modified to enable ML algorithms to utilize the input data.

### 3.6. Handling Imbalanced Data

This step aims to resolve any issues with a class imbalance in the dataset, especially if there is a wide gap in the distribution of the different classes.

Methods such as oversampling (such as SMOTE), undersampling, or class weight adjustment can address the disparity across classes. We do not perform any more operations to balance the imbalanced labels, even though we can see that our target variable’s labels are not evenly balanced nor significantly unbalanced.

#### Train–Test Split

The aim of this step is to distinguish the preprocessed dataset from the training and testing subsets. Using a stratified sampling approach guarantees that class distribution is retained across the training and testing sets. Using these pretreatment techniques, we can ensure that the dataset is prepared for future modeling and analysis. Remember to adapt and change these steps to our study’s unique requirements and characteristics as well as the CICIDS 2017 dataset.

The dataset is separated into training and testing using the train–test split method. The split dataset is displayed as follows:

x_train, x_test, y_train, y_test

Their shape is as follows:

(420040, 39) (105010, 39) (420040,) (105010,)

### 3.7. Exploratory Data Analysis

Engaging in exploratory data analysis (EDA) is necessary to comprehend the characteristics and patterns in the dataset. For our research on network traffic analysis utilizing the CICIDS 2017 dataset, we are able to employ the following crucial EDA techniques and visualizations.

#### 3.7.1. Class Distribution

We recommend utilizing either a bar chart or a pie chart to visually represent the distribution of class labels, such as distinguishing between usual traffic and different attack types. This visualization method allows us to better understand any class imbalance which is present and assists us in making informed decisions regarding preprocessing, including class balancing techniques.

**Figure 3:**
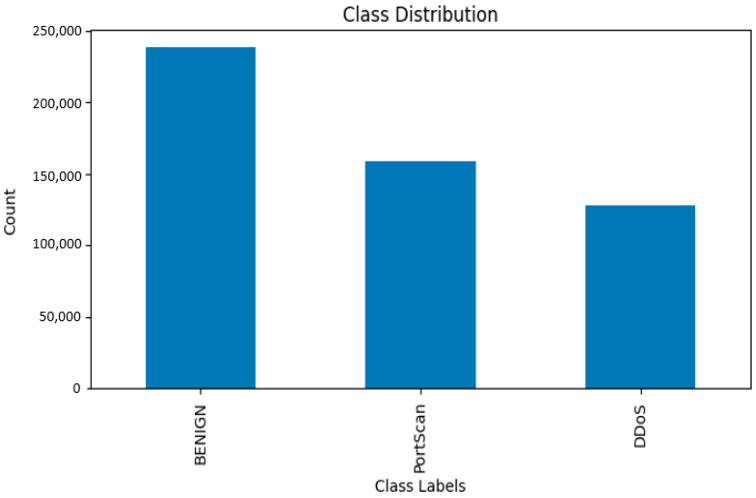
Class distribution of target variable.

The distribution of network traffic types is displayed in the depiction mentioned above (Figure 3). This illustrates that the maximum network traffic flow is categorized as normal, based on our existing network features. Conversely, network flows that do not fall under the normal category are further classified into two additional categories: Portscan (with a significant contribution) and DDos (the least strange contributor to the network traffic flow).

We recommend performing feature correlation analysis, which involves generating a correlation matrix or heatmap to visualize the relationships between various attributes. This analysis helps identify attributes with strong associations that may be crucial for the model’s performance.

Since there are nearly 75 features in our dataset, the image above demonstrates the correlation of a few chosen features. In this visualization, we displayed some features to determine the correlation between the forward packets, backward packets, and other features that are highly correlated, which are displayed in red. On the other hand, there is a weak correlation between the minimum and maximum packet lengths. It is also crucial to note how poorly connected most of the features are with one another.

**Figure 4:**
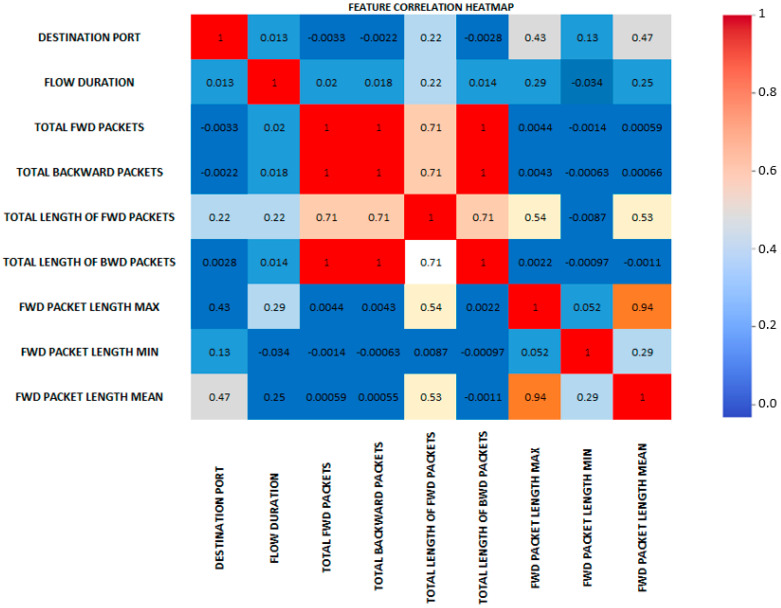
Correlation chart of selected features.

The feature correlation heatmap of the CICIDS 2017 dataset reveals the relationships and interdependencies between different attributes. For our network traffic analysis investigation, the visualization can show us the following information (Figure 4):

When two attributes exhibit a strong positive correlation (close to 1), they tend to increase or decrease together. Such attributes may contain redundant or similar information. In such cases, removing one of the related features is worth considering, and can reduce redundancy in the analysis.

A substantial negative correlation between two attributes (closer to −1) signifies an inverse relationship, implying that as one attribute increases, the other decreases. This information is valuable for comprehending network traffic flow and uncovering potential relationships between components.

The heatmap may help us to identify traits with minimal relationship to the target variable. These features may not be very predictive and may not significantly impact categorization. We might prioritize better correlation features when deciding which attributes are most important to train our network traffic analysis model.

The heatmap enables understanding the interactions between different features. We can discover more about the connections between the different traits by looking at the correlation patterns. This information can help to focus feature engineering efforts and give crucial context data for assessing model predictions.

Additionally, correlations might offer data for preprocessing methods. If two characteristics have a high positive or negative correlation, we can consider normalizing or standardizing them to ensure their scales are equivalent and prevent bias during model training.

We recommend generating histograms or boxplots depicting packet durations for different classes, including normal and assault categories. This approach allows us to gain insights into the distribution and potential variations, enabling us to identify patterns or specific characteristics associated with distinct types of traffic.

**Figure 5:**
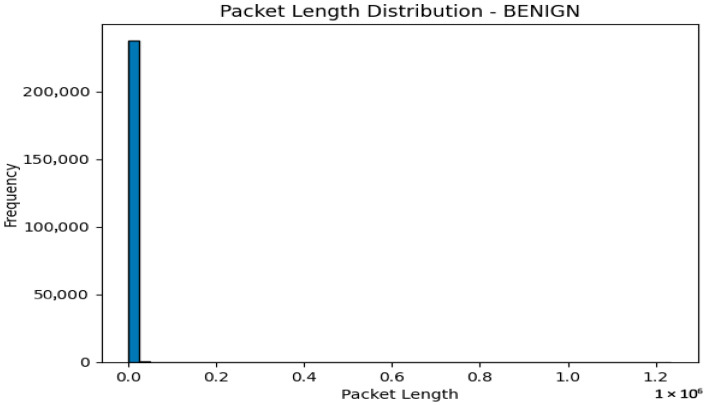
Packet length distribution of benign or normal traffic flow.

**Figure 6:**
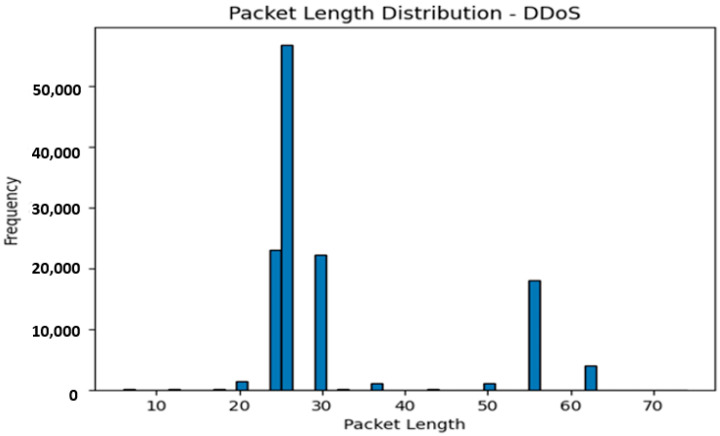
Packet length distribution of DDoS network traffic flow.

The packet length distribution of a benign or typical traffic flow is shown in Figure 5. In contrast, Figure 6 depicts the distribution of the DDoS network traffic flow’s packet lengths.

**Figure 7:**
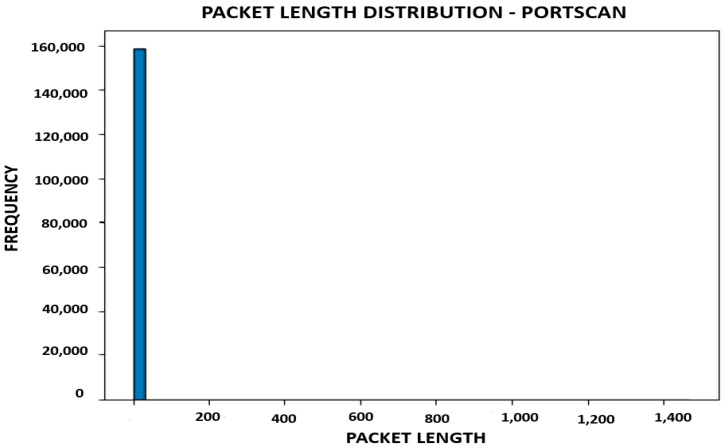
Packet length distribution of Portscan network traffic flow.

Figure 7 displays a histogram of the distribution of packet lengths for the selected traffic type. While the *x*-axis shows values for packet lengths, the *y*-axis shows the frequency or count of packets for each length. The visualization reveals the variation and distribution of packet lengths within the chosen network traffic class.

The analysis that results from displaying the packet length distribution can provide insight into the quirks of different types of network traffic. Here are some potential analyses that we could make:

#### 3.7.2. Identification of Traffic Patterns

By examining the packet duration distribution for each network activity type, we can identify any obvious trends or fluctuations in packet length. For instance, it depicts a typical pattern for ordinary network traffic if the “regular” traffic class distribution peaks around a specific packet length range.

On the other hand, if some attack classes exhibit atypical or anomalous packet length distributions, it implies that differences in packet lengths are related to specific assaults.

#### 3.7.3. Differentiation between Network Activities

We can distinguish between genuine and malicious network activity by contrasting the packet length distributions across different traffic classifications. If the distributions are noticeably different, packet length may be valuable for identifying various types of network traffic.

The graphic makes it easier to quantify and visually assess the differences in packet lengths between different classes.

- Flow Duration: This is used to visualize the distribution of flow durations for the different classes using boxplots or violin plots. It can demonstrate whether particular traffic classes exhibit observable flow length patterns.

**Figure 8:**
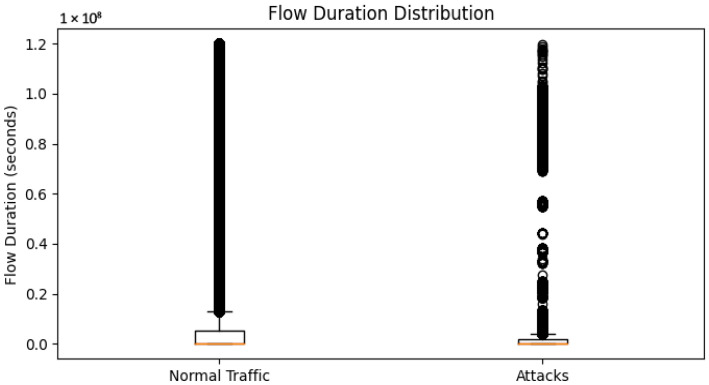
Flow duration of normal and anomaly traffic flow distribution.

The duration of the normal and abnormal traffic flow patterns is shown in Figure 8.

This method separates the data into two subsets based on the class labels: normal traffic and attacks. Then, it generates boxplots showing the flow duration distribution for regular traffic and assaults using the “Flow Duration” function. Boxplots show the median, quartiles, and any outliers in the data.

The *y*-axis displays the flow time in seconds, and the *x*-axis displays the two types of traffic: “normal traffic” and “attacks”. Each category’s boxplot displays the flow duration distribution and any variations between ordinary traffic and assaults.

Interpreting the flow duration distribution graphic can provide the following insights:

- The median value, or line inside the box, represents the central tendency of the flow durations for each group. By contrasting the medians of regular traffic and assaults, it is possible to identify potential changes in flow length patterns.
- Interquartile Range (IQR): The box shows the quartiles, which are the middle 50% of the data, as the interquartile range (IQR). The distribution of flow durations within each category is shown by the lower and upper quartiles, Q1 and Q3, respectively.
- Outliers, or data points that stand out from the others, are those outside the whiskers (lines extending from the box). These indicators of anomalous or unusual flow lengths demand further study.
- By examining the flow duration distribution using boxplots, we can learn more about the differences in flow durations between normal traffic and attacks. This image helps distinguish between safe network traffic and malicious activities and shows potential flow duration patterns specific to different classifications.
- Dimensionality Reduction Visualization: Utilize dimensionality reduction techniques, such as the t-SNE (t-Distributed Stochastic Neighbor Embedding) or PCA (principal component analysis), to visualize the high-dimensional feature space in a lower-dimensional scatter plot. Color the spots according to their respective class names to visualize the clustering or separation of different traffic categories.

This approach uses principal component analysis (PCA) to reduce dimensionality and compress the feature space into three dimensions. PCA separates the features from the target variable (“label”) to convert the feature matrix X into an X_pca with three major components.

**Figure 9:**
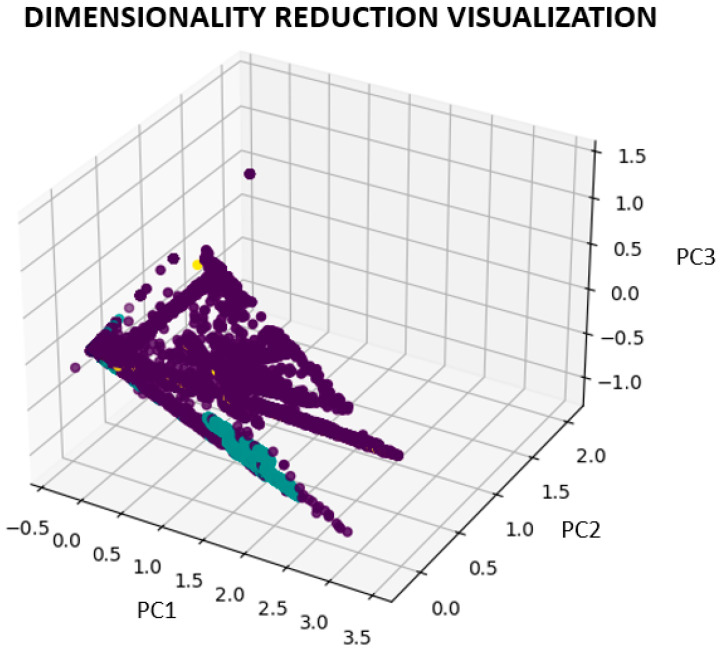
Dimensionality reduction visualization of features.

The algorithm then creates a three-dimensional scatter plot to display the data in compressed dimensions. Each dot on the plot represents a dataset instance and is colored according to the class labels (“label”) (Figure 9).

#### 3.7.4. Interpreting the Dimensionality Reduction Visualization

This subsection will explore the interpretation of dimensionality reduction visualizations created using techniques such as PCA. These visualizations offer valuable insights into various aspects of our data. Specifically, the following:

- Class Division: The visualization allows us to understand the division or grouping of instances from different classes. If instances from distinct classes are separated in reduced dimensions, the features used in PCA effectively distinguish between the classes.
- Classes that Overlap or are Closely Interleaved: This suggests that it may be challenging to distinguish the classes based on the selected attributes. It happens when examples from several classes are closely interspersed or overlayed in the reduced dimensions. Additional feature engineering or more sophisticated dimensionality reduction techniques may be required.
- Patterns and Structures: When compared to the original high-dimensional space, the visualization may make patterns or structures in the data that were previously hidden obvious. For example, we could identify clusters, outliers, or linear or non-linear connections between the occurrences.

By using dimensionality reduction and PCA to visualize the data, we can determine the structure and separability of the CICIDS 2017 dataset in a low-dimensional space. Further research or modeling decisions can be aided by understanding the data distribution, detecting probable patterns or clusters, and using this visualization.

### 3.8. Network Traffic Analysis

Network traffic analysis examines and interprets network traffic data’s patterns, behaviors, and characteristics. It involves analyzing the packet traffic within a network to discover more about network activity, identify anomalies, and improve network performance and security.

Network traffic analysis can be used to learn about several aspects of network communication, including the source and destination of data packets, the protocols being used, packet sizes, timestamps, and other important metrics. By examining this data, network managers and security analysts can identify patterns in network use, identify suspicious or malicious activities, and maximize the use of network resources.

The basic objectives of network traffic analysis are as follows:

- Monitoring Network Performance: Managers can gain additional insight into performance metrics, including bandwidth consumption, traffic congestion, latency issues, and others, by looking at network traffic. It facilitates resource efficiency and the simplification of network operations.
- Identifying Potential Security Threats: Network traffic analysis is crucial for identifying anomalies and intrusions, such as attempted intrusions, malware infections, and unauthorized access. Spotting suspicious activity and putting the necessary security measures in place by looking at network traffic patterns and spotting variations from typical behavior is possible.
- Debugging and Troubleshooting: Traffic analysis can help identify the root cause of network problems and aid debugging and troubleshooting. By examining network traffic flows, administrators can locate areas of congestion, improper configurations, or damaged equipment, enabling efficient troubleshooting and remedies.

Traffic engineering and capacity planning are aided by network traffic analysis, which provides data on traffic patterns and trends. Having a firm knowledge of the volume and nature of network traffic allows administrators to allocate resources, update hardware, and enhance network performance.

Network traffic analysis is crucial for enterprises as it supports compliance with regulatory standards and requirements. This involves monitoring network usage, identifying policy violations, and ensuring adherence to privacy and data protection legislation.

Network traffic analysis is crucial for both network administration and security. Examining network traffic data is necessary to comprehend network activity, identify anomalies, boost performance, and ensure the overall integrity and security of the network infrastructure.

### 3.9. Deep Neural Decision Forest (DNDF)

The benefits of deep neural networks and decision forests are combined in an ML model called the DNDF. Deep neural networks for learning features are fused with decision forests for decision-making and ensemble learning.

**Figure 10:**
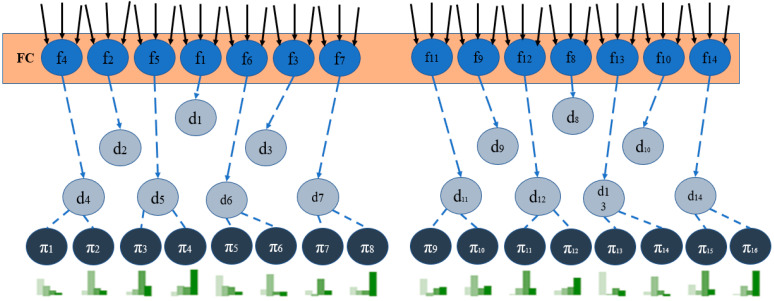
DNDF model architecture implementation.

Deep neural networks that can be used to learn features from the DNDF include the CNN and feed-forward neural networks (FNNs). The neural network is trained on the input data to get meaningful representations or features from the raw input. Following feature learning, the DNDF incorporates decision forests, merely collections of decision trees. Each decision tree in the forest makes predictions based on a subset of the learned attributes. The decision trees are trained to segment the feature space and provide predictions using rules and thresholds (Figure 10).

A DNN with a variable layer count and representation with parameters at the top. Block F.C.: The fully connected layer offers the following functions, which are defined in Equation (1).

dn(x; Θ) = σ(fn(x; Θ))
(1)

The routing (split) choices dn(x) = (fn(x)) are created when each output of fn is brought into correspondence with a split node in a tree. It is possible to add output units to decision nodes in any sequence (the arrangement we describe provides for a straightforward visualization). As a result of resolving the convex optimization issue described in Equation (1), the bottom circles are leaf nodes containing probability distributions.

We now consider the minimization concerning π when Θ is fixed. This is represented by Equation (2).

minπR(Θ,π; T)
(2)

DNDF combines the forecasts given by the decision trees in the forest to produce the final forecast. It may combine every decision tree forecast using ensemble techniques, such as weighted averaging or majority voting.

The DNDF model aims to exploit the interpretability and ensemble learning of decision forests as well as the representational learning potential of deep neural networks. It can identify complex patterns and correlations in the data by combining a deep neural network with the diversity and robustness of a decision forest ensemble. The DNDF model’s decision forest and deep neural network components are typically trained separately before being integrated for inference during implementation. The deep neural network is trained on the input data using common optimization techniques such as backpropagation and stochastic gradient descent. The decision forest is trained using methods such as the random subspace technique, bootstrapping, or random forests. To implement the DNDF model for network traffic analysis using the CICIDS 2017 dataset, we would need to tweak it to fit the particular requirements of our research and dataset. It can necessitate changing the architecture, hyperparameters, and training technique, depending on the peculiarities of the network traffic data and the classification task at hand.

## 4. Methodology

**Figure 11:**
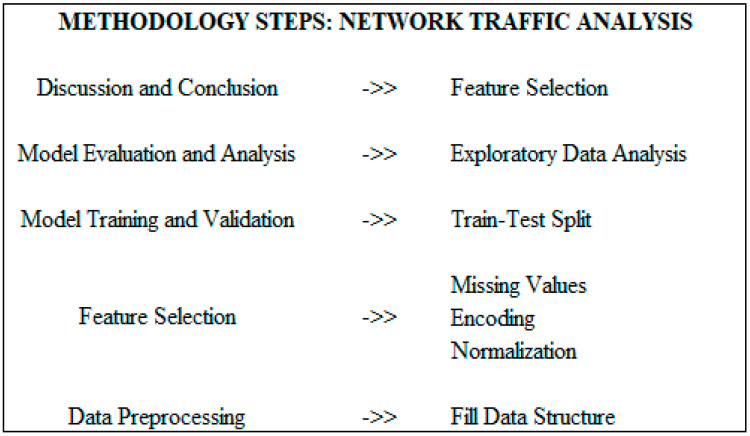
Methodology steps for network traffic analysis using DNDF.

The following is a high-level method for performing a network traffic analysis using the DNDF model on the CICIDS 2017 dataset and is also depicted in Figure 11 as follows:

### 4.1. Data Preprocessing

Incorporating the CICIDS 2017 dataset into an appropriate data structure, such as a Pandas data frame, is advisable. Vital data preparation steps encompass handling missing values, encoding categorical variables, and normalizing numerical features. To assess the effectiveness of the DNDF model, it is essential to partition the dataset into training and testing sets.

We prioritize data integrity throughout the process, ensuring it is well-structured within a Pandas data frame. Continuously, we vigilantly monitor for shifts in feature distributions, detect missing values, and remain attentive to changes in data quality. It is crucial to regularly update the training and testing datasets to adapt to evolving network traffic patterns.

Incorporating the CICIDS 2017 dataset into a suitable data structure, such as a Pandas data frame, is highly recommended. This entails essential steps such as handling missing values, encoding categorical variables, and normalizing numerical features. Further, dividing the dataset into training and testing sets allows us to assess the DNDF model’s performance effectively.

We emphasize the importance of unwavering diligence in overseeing input data throughout the analysis. This involves maintaining its structure within a Pandas data frame and remaining vigilant for any shifts in feature distribution, missing data instances, or data quality alterations. The consistent updating of both training and testing datasets is imperative to accommodate the dynamic nature of network traffic patterns.

### 4.2. Feature Selection and Engineering

Use exploratory data analysis to understand attributes’ distribution and the dataset, and to find pertinent linkages. Use feature selection techniques such as correlation analysis or mutual information to identify the most important features for network traffic analysis.

Consider domain expertise and professional insights while designing extra features that could improve the DNDF model’s functionality.

### 4.3. Model Training and Validation

The DNDF architecture, which combines decision forest and DNN elements, should be created and configured. The training dataset, appropriate optimization methods, and loss functions are used to train the deep neural network. The learned deep neural network’s recovered features are used to train the decision forest component. Recall, accuracy, precision, and F1-score are suitable evaluation metrics for evaluating the DNDF model’s performance on the testing dataset.

Using cross-validation or other techniques, evaluate the robustness and generalizability of the model.

### 4.4. Mathematical Model

The decision forest is a collection of decision trees. Each decision tree is constructed via recursive partitioning, according to the splitting criteria that categorize the data into groups. The splitting criterion may also be calculated using other metrics, such as information gain, the Gini index, or entropy. The decision trees in the forest collectively predict outcomes by averaging the outputs of individual trees [46]. DNNs are composed of artificial neurons coupled in several layers and are frequently called nodes or units. Each neuron applies a non-linear activation function to the weighted sum of its inputs. The weights connecting the neurons are learned using backpropagation, which involves iteratively changing the weights by the gradient of a loss function about the network parameters. During training, the deep neural network approximates the basic mapping between the input features and the target labels.

The DNDF model combines decision forests with deep neural networks to use their complementing features. The decision forest component provides interpretability and feature significance analysis, while the deep neural network component enables the model to capture complex patterns and non-linear correlations in the data.

The input features are represented by x in the DNDF model, which will be written as f(x).

#### 4.4.1. Decision Forest Component

F(x), which is an ensemble of decision trees, may be used to represent the decision forest. The denotation of each decision tree in the forest is T_i(x), where ‘i’ stands for the tree’s index. It is possible to compute the decision forest component F(x)’s output as Equation (3) as follows:

F(x) = 1/N × (T_i(x))
(3)

In this case, N represents the overall number of decision trees in the forest and the total number of decision trees.

#### 4.4.2. Deep Neural Network Component

The deep neural network, which consists of several layers of linked neurons, may be expressed as DNN(x). N_j, where j is the neuron’s index, can be used to represent each neuron. It is possible to compute the output of the deep neural network component DNN(x) by using Equation (4).

DNN(x) = N_L(N_L − 1(…N_2(N_1(x))))
(4)

In this case, L stands for the total number of layers in the network, and N_i stands for the output of layer i.

#### 4.4.3. Integration of Decision Forest and Deep Neural Network

The final prediction is created by combining the outputs of the deep neural network component DNN(x) with the decision forest component F(x).

The DNDF model’s ultimate output may be computed using Equation (5).

F(x) = F(x) + DNN(x)
(5)

Here, we see the weighting coefficients that establish how much each component contributes to the outcome of the prediction. These coefficients can be changed depending on the desired ratio between the decision forest and the deep neural network.

### 4.5. Model Architecture Design

**Figure 12:**
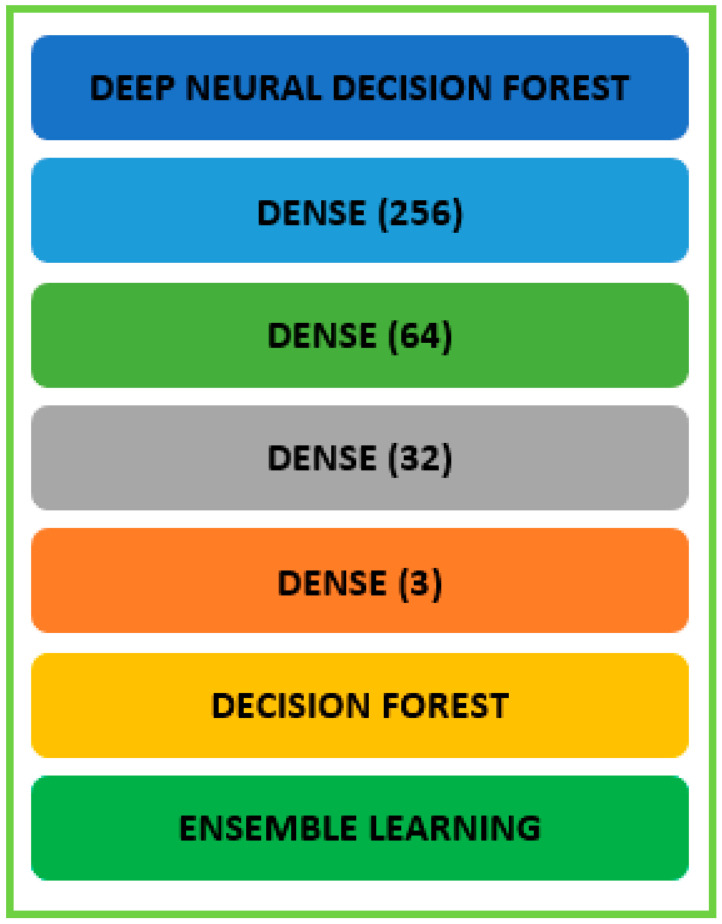
Model architecture design of proposed DNDF model.

DNDF model architecture design (Figure 12) for network traffic analysis utilizing the CICIDS 2017 dataset is shown below.

#### Deep Neural Network (DNN) Component

The structure of the DNN model includes the following components:

- Input Layer: The input layer obtains the dataset’s characteristics related to network traffic. The dimensionality of the input characteristics affects how many neurons are present in this layer.
- Hidden Layers: Several hidden layers might be added to identify and understand intricate patterns in the data. Depending on the type of network traffic data, we may utilize a variety of layer types, such as fully connected (dense), convolutional, or recurrent layers. Each hidden layer usually uses an activation function to introduce non-linearity and helps the model learn non-linear correlations. Sigmoid, tanh, and ReLU (Rectified Linear Unit) are typical activation functions.
- Dropout and Regularization: We may use regularization methods such as L1 or L2 regularization or dropout layers to avoid overfitting.
- Output Layer: The anticipated class probabilities are represented by the output layer. The number of classes in the network traffic classification task corresponds to the number of neurons in this layer. Softmax activation may be used to get class probabilities.

We emphasize the importance of considering model architecture design. In response to the evolving nature of network traffic, we conduct extensive testing on the DNN component’s structure. This testing encompasses various settings, including the number of layers, neurons, and activation functions. Additionally, we incorporate regularization and dropout techniques to mitigate overfitting, particularly as the dataset’s properties undergo changes.

### 4.6. Decision Forest Component

The decision forest component comprises a group of decision trees, each trained to generate predictions using a subset of characteristics obtained from the DNN component. Leaf nodes indicate class predictions, while interior nodes reflect feature tests in each decision tree. To generate the final forecast, the predictions from several decision trees in the forest can be integrated using techniques such as majority voting or weighted averaging.

### 4.7. Integration of DNN and Decision Forest

The deep neural network component extracts significant features from the network traffic data. A subset of pertinent characteristics for the decision forest component is chosen or changed from the retrieved features. Training and Inference: The DNN component is trained individually using backpropagation and optimization methods. The decision forest component then receives the newly learned features for training and inference. Model Fusion: To reach the final forecast, the predictions from the DNN and decision forest components may be fused or blended.

TensorFlow or PyTorch are two examples of deep learning frameworks that can be used to create the model architecture. Based on the properties of the CICIDS 2017 dataset and the particular network traffic analysis task, the precise configuration, including the number of layers, neurons, activation functions, and regularization approaches, may be customized and optimized.

### 4.8. Model Training

#### CICIDS 2017 Dataset

**Figure 13:**
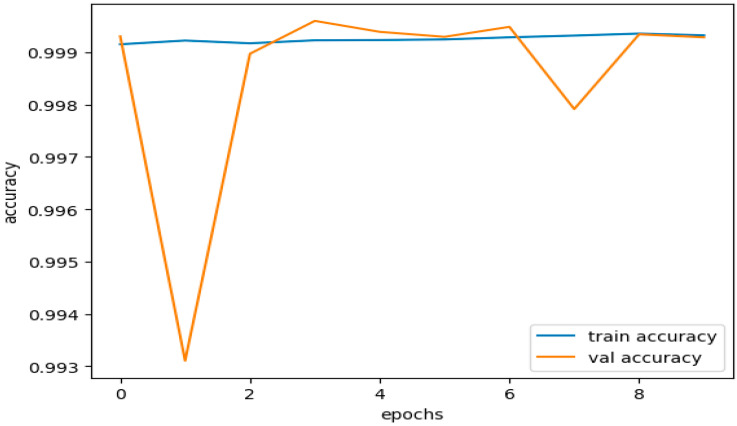
Training and validation accuracy performance of the training subset.

According to the training results, the model had a high training accuracy of 0.9993 and a low training loss of 0.0033. It is reflected in the validation results provided in Figure 13, which show a low validation loss of 0.0035 and a high validation accuracy of 0.9993.

**Figure 14:**
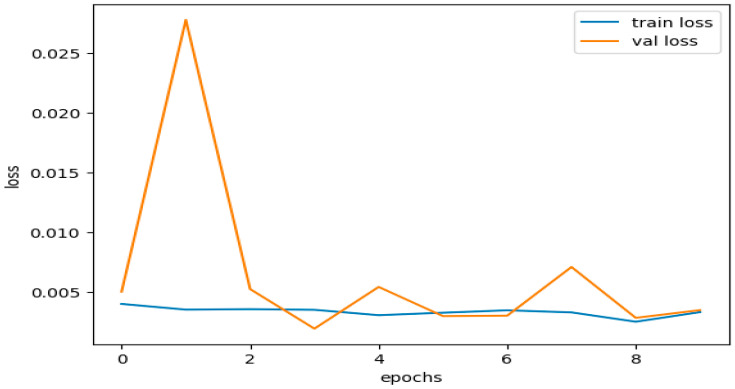
Training and validation loss performance of the training subset.

These results suggest that the model completed both training and validation. The model successfully minimized the gap between the predicted outputs and the actual labels in the training and validation datasets, as shown by the small training and validation losses presented in Figure 14. This demonstrates how well the model and training set fit each other.

High accuracy rates during training and evaluation are proof that the model works. A high accuracy score means the model correctly forecasted most cases in both the training and validation datasets. The results demonstrate that the model effectively generalized the training data to the unseen data, comprehending the fundamental trends and relationships within the dataset.

The consistent training and validation results show that the model did not demonstrate overfitting or underfitting. Overfitting, which hinders a model’s capacity to generalize to new data, occurs when a model becomes excessively complex and starts to recall the training set. On the other hand, underfitting occurs when the model fails to capture the underlying patterns in the data and performs badly on both the training and validation datasets.

Based on the strong agreement between training and validation measures, including loss and accuracy, the model was effective in striking a good balance between fitting the training data and generalizing it to unknown data. It demonstrates that the model is accurate and well-optimized in classifying situations in the dataset.

## 5. Model Evaluation

**Figure 15:**
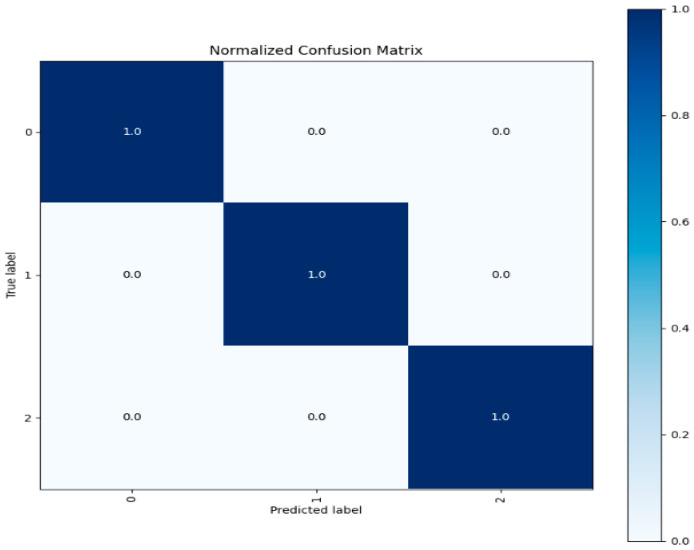
Confusion matrix of three label classes.

We ran a model evaluation to assess how well our trained model fared in our research. Using a range of assessment metrics, we assessed the model’s effectiveness in classifying instances of network traffic (Table 11). The following are the often-used evaluation metrics and their definitions:

- Accuracy: The percentage of cases in the dataset that were properly categorized is a frequently used metric. It offers a comprehensive evaluation of the model’s predictive power. Equation (6) illustrates this metric.
Accuracy = total number of predictions/number of correct predictions
(6)
- Precision: Precision is the proportion of all correctly classified cases or true positive predictions that the model made out of all positive predictions. It focuses on the precision of optimistic forecasts and aids in assessing how effectively the model can prevent false positives. Equation (7) illustrates this metric.
Precision = true positives + false positive/true positives
(7)
- Recall (Sensitivity or True Positive Rate): Recall calculates the percentage of true positive forecasts based on all of the dataset’s real positive cases. It focuses on how well the model can spot good cases and avoid false negatives. Equation (8) illustrates this metric. Equation (8) illustrates this metric.
Recall = true positives + false negatives/true positives
(8)
- F1-Score: The F1-Score is a harmonic measure of recall and accuracy. It provides a fair evaluation of recall and accuracy that considers both false positives and false negatives. The F1-Score is highly useful when there is a mismatch between the classes in the dataset. Equation (9) illustrates this metric.
F1-Score = precision + recall/2 × precision × recall
(9)
- Confusion Matrix: A confusion matrix is a table that shows how well a classification model works by comparing expected labels to actual labels. It provides in-depth data on true positives, false positives, and false negatives to allow for a more thorough evaluation of the model’s performance. Figure 15 depicts the confusion matrix of three label classes.

Each test entry is correctly predicted for each class according to the confusion matrix visualization for each class shown above. Three different patterns of network traffic flow existed. One was labeled as typical network traffic flow, and the other as oddities. The test data for each class exhibited accurate predictions, confirming the model’s reliability and its ability to generalize to previously unobserved data. We evaluated our model’s capability to classify network traffic instances by accurately using various assessment criteria. These criteria include overall accuracy, F1-Score, recall, and precision metrics. Additionally, the confusion matrix helps to pinpoint particular strengths and weaknesses in the model’s predictions, as illustrated in Figure 15.

**Figure 16:**
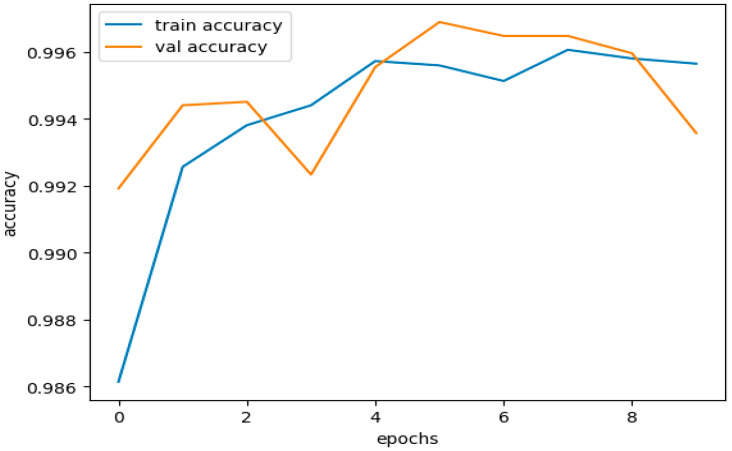
Training and validation accuracies of the testing subset.

**Figure 17:**
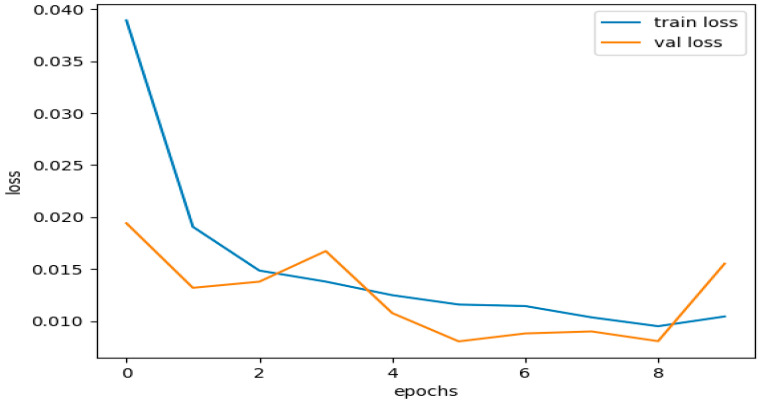
Training and validation losses of the testing subset.

Figure 16 illustrates the training and validation accuracies for the testing subset of the CICIDS 2017 dataset. Figure 17 presents the curves depicting the training and validation losses for the same testing subset of the CICIDS 2017 dataset.

Next, we proceed by dividing this data into training and testing subsets, resulting in the following sizes:

X train = (38614, 11).
X test = (9654, 11).
Y train = (38614).
Y test = (9654).

The dataset was trained using the same DNDF model and hyperparameters, and the results are displayed in Figure 16 and Figure 17, and shows an accuracy of 0.9968 and a loss of 0.0104. The obtained validation accuracy and loss were 0.0155 and 0.9936, respectively.

The performance of our model is shown in Table 12 for both the training and unseen validation datasets, demonstrating that neither overfitting nor underfitting affects the model. We used a testing dataset to deduce evaluation measures and their corresponding curves.

**Figure 18:**
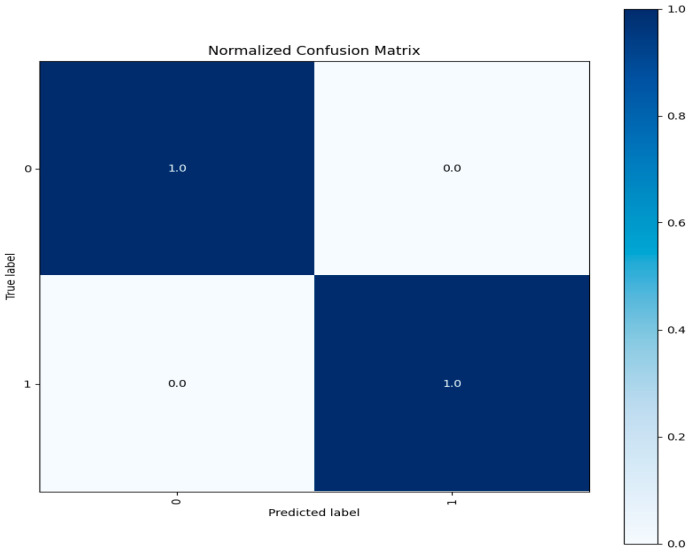
Confusion matrix for unseen test data of the CICIDS 2017 dataset.

The evaluation metrics’ performance on the unseen test data is highly satisfactory, exhibiting a performance level similar to that observed in the CICIDS 2017 dataset. The associated confusion matrix is presented below in Figure 18.

The test data demonstrates accurate predictions for both attack and normal classes, with no misclassified points. This emphasizes the exceptional performance of our model architecture, which achieves a near-identical 99% accuracy rate, while the other evaluation metrics also demonstrate satisfactory results.

### 5.1. DNDF Model PERFORMANCE Using the CICIDS 2018 Dataset

For this dataset, we have not extracted more features since this dataset already has more comprehensive features. The dataset is split into training and testing subsets as follows:

X train = (835800, 78).
X test = (208951, 78).
Y train = (835800).
Y test = (208951).

This dataset is trained on the same model with the same hyperparameters and achieved a training accuracy of 1.0000 and a training loss of 6.2132 × 10^−4^. DNDF achieved 1.0000 and 5.2341 × 10^−4^ as validation accuracy and loss, respectively.

It is worth emphasizing that the model achieved higher accuracy and significantly lower loss values for the CICIDS 2018 dataset. This outcome underscores the notion that the comprehensiveness and size of the dataset influence our model’s performance. Below, you will find the training and loss performance curves obtained when using the CICIDS 2018 dataset.

**Figure 19:**
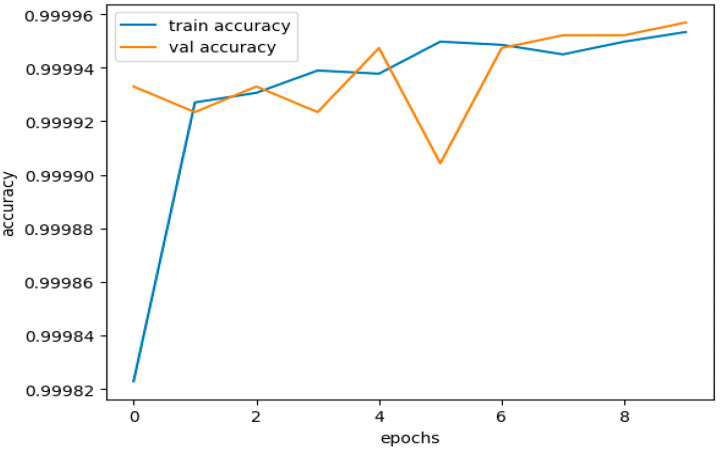
Training and validation accuracies using the CICIDS 2018 dataset.

**Figure 20:**
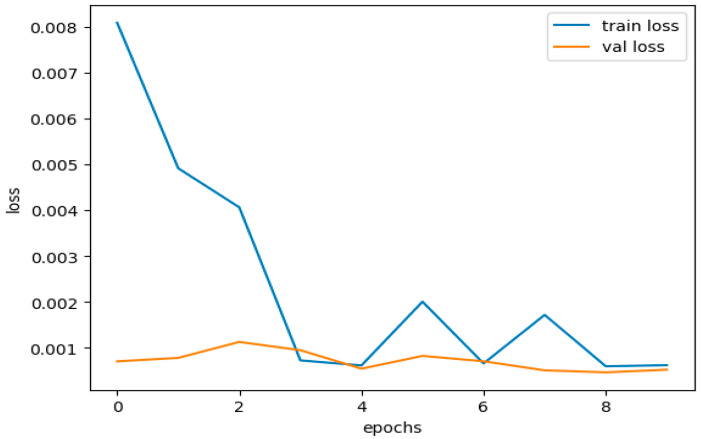
Train and validation losses using the CICIDS 2018 dataset.

Figure 19 illustrates our model’s training and validation accuracies using the CICIDS 2018 dataset. Figure 20 presents the curves depicting the training and validation losses.

The performance curves above indicate that the accuracy and loss curves for the training and validation datasets showed satisfactory behavior. Although there were minor fluctuations in the training curves, they did not reach significant levels. These fluctuations, within acceptable limits, prove that our model was trained effectively without any signs of overfitting or underfitting.

For the CICIDS 2018 dataset, the performance metrics’ values of our DNDF model are provided in Table 13.

The table above demonstrates that the model performs exceptionally well on unseen data, excelling not only in terms of accuracy but also across various evaluation metrics.

**Figure 21:**
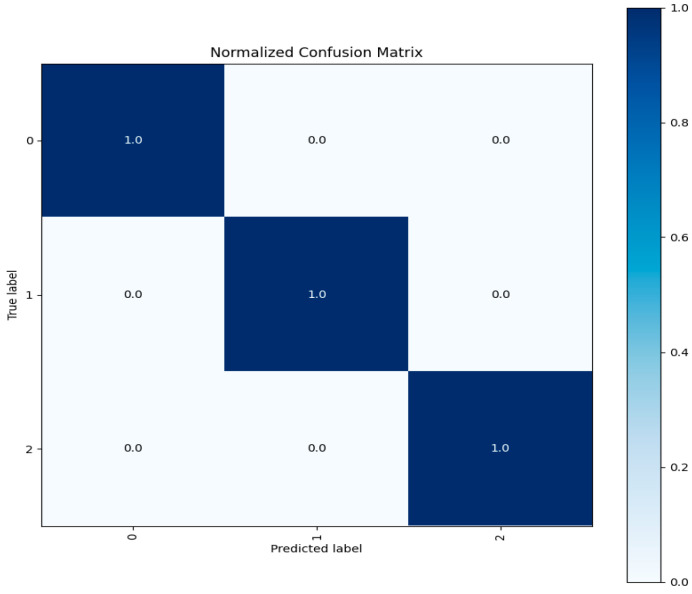
Confusion matrix for testing data used from the CICIDS 2018 dataset.

Figure 21 shows the confusion matrix for testing data used from the CICIDS 2018 dataset.

Here, we now have three classes compared to two in the custom dataset. It should be noted that all classes are accurately predicted, with no misclassifications in the test data. This finding highlights the DNDF model’s remarkable performance across several datasets.

By analyzing our DNDF model’s performance on multiple datasets, we broadened its potential. In the first scenario, we used the CICIDS 2018 dataset, an expanded version of the dataset previously used. Notably, the model performed better than the CICIDS 2017 dataset, obtaining an accuracy of 100% as opposed to the latter’s accuracy of 0.9993. Additionally, the loss for the 2017 dataset was 0.0027, but it was 6.2132 × 10^−4^ for the 2018 dataset. These minor performance variations demonstrate our DNDF model’s remarkable capabilities and consistency across datasets with similar architecture and hyperparameters.

Wireshark and Metasploit, two network analysis tools that operate well together, were used to create a new dataset. The network traffic of connected systems, including Bluetooth, Wi-Fi, and Ethernet cable interfaces, was properly examined with Wireshark. We used Metasploit, a tool that simulates various attacks within the host system, to promote dataset comprehensiveness. Both XSS and SQL injection were incorporated into the simulated attacks. Wireshark was used to analyze and log the resulting network traffic meticulously. We meticulously extracted additional protocol, flow, and packet-level features to enrich the generated dataset further. After feature extraction, we labeled the dataset and trained the DNDF model using consistent hyperparameters.

DNDF yielded an accuracy of 0.9956, which is below that obtained with the CICIDS 2017 and CICIDS 2018 datasets, yet still exhibited remarkable performance. Furthermore, the model achieved a loss of 0.014. We thoroughly evaluated the DNDF model’s performance through this systematic experimentation, yielding consistently satisfactory results.

### 5.2. Proposed Model Design

**Figure 22:**
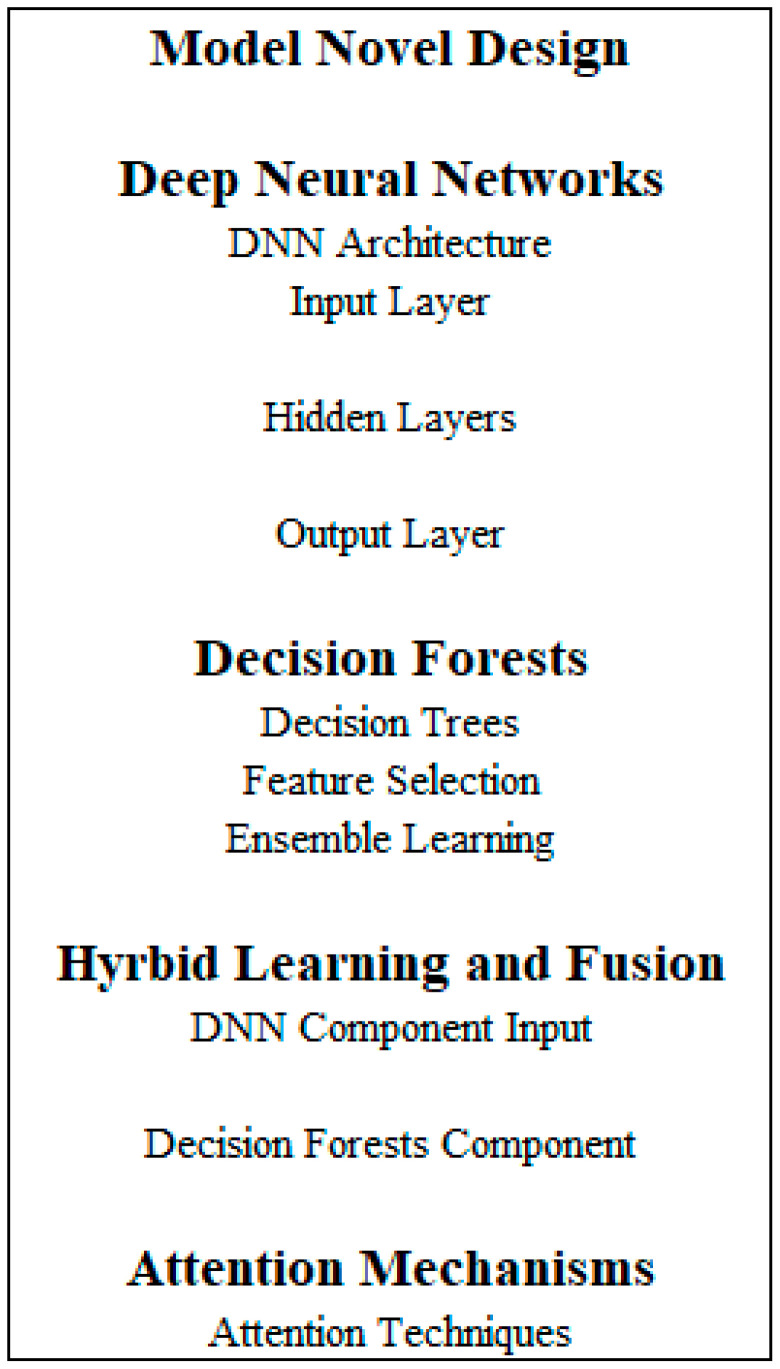
Model architecture and workflow.

Our research proposes a novel DNDF approach for accurate network traffic analysis using the CICIDS 2017 dataset, as shown in Figure 22. DNN and decision forests are coupled in DNDF to achieve precise and reliable traffic categorization.

- DNNs: We use DNNs as the DNDF architecture’s basic model. Being strong models, DNNs can learn complex representations from raw input data. To find complicated correlations and patterns in network traffic data, our technique builds a deep architecture composed of numerous layers of neurons.
- Decision Forests: Besides DNNs, we include decision forests in the DNDF architecture. Decision forests, which are ensemble learning models, are created by combining several decision trees. Each decision tree is trained using different traits or data samples to encourage diversified and complementary learning. A reliable and accurate traffic classification can be produced using projections from many decision trees.
- Hybrid Learning and Fusion: To leverage the advantages of both decision forests and DNNs, the DNDF technique uses a hybrid learning and fusion process. To learn hierarchical representations of the input data, the DNN component of the model first gets network traffic data. The decision forest component performs classification using the learned representations as the input and an ensemble of decision trees. The forecasts from the two components are combined to produce the outcome.
- Attention Mechanisms: To improve the model’s capacity for discrimination, we incorporate attention mechanisms into the DNDF framework. Attention approaches allow the model to dynamically focus on the most instructional features or chunks of the traffic data throughout the categorization phase. The model may then give more weight to important features, improving the prediction’s overall accuracy and understandability.

### 5.3. Model Optimization and Regularization

We employ a range of optimization and regularization techniques to ensure the model’s usability and generalizability. It covers techniques, such as batch normalization, dropout, and weight decay, that reduce overfitting and improve model robustness.

By combining the benefits of deep neural networks, decision forests, attention mechanisms, and model regularization techniques, the DNDF methodology offers a novel and effective method for accurate network traffic analysis. To obtain outstanding classification accuracy on the confronting CICIDS 2017 dataset, decision forests’ ensemble learning capabilities are combined with the DNN’s rich representations.

#### Core Contributions

**Figure 23:**
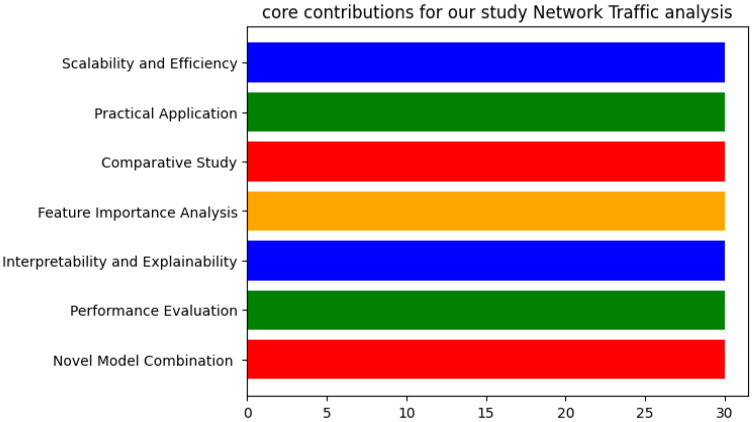
Core contribution of our study network traffic flow analysis.

The following points presented in Figure 23 show the essential contributions to our study on network traffic analysis using the DNDF model:

- Novel Model Combination: For network traffic analysis in particular, the paper suggests including decision forests and DNNs in the DNDF model. The advantages of both elements are used in this article’s new and effective way for precise categorization and interpretability.
- Performance Evaluation: The study comprehensively evaluates the DNDF model’s performance using the CICIDS 2017 dataset. It assesses the model’s accuracy, precision, recall, and F1 score by comparing it to other recent models or baseline methods. This contribution demonstrates the DNDF model’s ability to classify network traffic appropriately.
- Interpretability and Explainability: This study concentrates on the interpretability and explainability of the DNDF model’s results. Examining the decision rules and feature importance generated by the decision forest component offers knowledge of the factors influencing the categorization decisions. This information advances our comprehension of network traffic patterns and aids in detecting malicious or anomalous conduct.
- Feature Importance Analysis: Each character in the DNDF model is carefully analyzed to determine its importance. To properly categorize network traffic, it identifies its fundamental components. By giving knowledge about the key signs of network attacks or irregularities, this contribution helps in the development of efficient intrusion detection systems.
- Comparative Study: By using different State-of-the-Art models or techniques for network traffic analysis, the performance of the DNDF model is compared. While stressing its advantages over other tactics, it evaluates the model’s accuracy, robustness, and effectiveness. This article focuses on the distinction and superiority of the DNDF model in the context of network traffic analysis.
- Practical Application: This study discusses the potential applications of the DNDF model to real-world issues, including network security and anomaly detection. The possible benefits and outcomes of the DNDF paradigm for proactive network monitoring and response are highlighted. This contribution demonstrates the DNDF model’s usefulness in enhancing network security.
- Scalability and Efficiency: This study examines the DNDF model’s scalability and efficiency, particularly when handling huge network traffic datasets. We examine the model’s memory usage, training and testing times, and computing requirements. This contribution helps determine whether the DNDF model is applicable in high-throughput or real-time network scenarios.

These key contributions demonstrate the novelness, power, and utility of using the DNDF model for network traffic analysis. They advance network security and intrusion detection methods by providing knowledge of the model’s usability, interpretability, feature importance, and comparative advantages.

### 5.4. Comparative Analysis

We compared our proposed DNDF method in this study to the 2D-CNN, Xgboost, and RF (random forest) reference methods, as shown in Table 14. Each approach was evaluated using the CICIDS 2017 dataset, and the accuracy measure was used for comparison.

The first reference method, 2D-CNN, obtained an accuracy of 90.6% on the CICIDS 2017 dataset. This approach aims to detect malicious network traffic with the least amount of preparation work. The achieved precision was significantly lower than other techniques, which must be considered. The drawbacks of the 2D-CNN approach may be due to the model design, feature representation, or optimization techniques that were employed.

The second reference method, Xgboost, achieved an accuracy of 99.7% on the CICIDS 2017 dataset. This method classifies network traffic using statistical description sampling and hierarchical ensemble learning. While it shows promise and achieves accuracy comparable to the 2D-CNN method, there may be room for improvement in how it manages complex patterns and extracts sufficient information from network traffic data.

The third reference method, RF (random forest), performed better than the first two methods on the CICIDS 2017 dataset, with an accuracy of 99.9%. RF uses ensemble learning capabilities to obtain high traffic flow-based intrusion detection accuracy. However, it is crucial to remember that RF relies on decision trees as its underlying models, and might need help to completely capture complex interactions and non-linear correlations in the data.

Our DNDF method outperformed the reference approaches on the CICIDS 2017 dataset, achieving an accuracy of 99.96%. Collaboration between deep learning and decision trees, enhanced feature representation, model optimization techniques, and robustness to noisy and imbalanced data are all aspects that helped this progress. These factors helped DNDF to achieve great performance in precisely detecting instances of network traffic, making it a useful model for network traffic analysis.

## 6. Discussion

The DNDF model for network traffic analysis was trained and evaluated using the CICIDS 2017 dataset in this research project. Using the dataset, we were able to conduct research on the features and patterns of network traffic flows, determine whether or not there were any potential risks or anomalies, and construct a reliable classification model. In this study, we extensively used the features offered by the CICIDS 2017 dataset to conduct an in-depth investigation of network traffic and accurately categorize the many kinds of activities that might occur in a network. This dataset made a significant contribution to the development of intrusion detection and network traffic analysis, as well as to the validity and reliability of our study. In addition, our study was conducted with it.

We recommend a two-layer structure combined with a CNN and the decision forest for the classification module. When producing the final forecast, the DNDF considers all of the predictions made by the decision trees in the forest. It can integrate the predictions of each decision tree by employing many different ensemble procedures, such as weighted averaging or majority voting. The purpose of the DNDF model is to use the interpretability and ensemble learning capabilities of decision forests in addition to the possible representational learning capabilities of DNN. As it combines a deep neural network with the diversity and robustness of a decision forest ensemble, it can recognize intricate patterns and correlations hidden within the data. If we were to analyze network traffic using the same dataset as before using the DNDF model, we would need to modify the model to satisfy the specific needs of both our research and the dataset. It may be necessary to modify the architecture, the hyperparameters, and the training technique. The specifics of the network traffic data and the classification task at hand play a role in this decision. The model was evaluated to determine how well our previously trained model performed in this research. We evaluated the model’s efficacy in identifying instances of network traffic by using a variety of assessment metrics, such as recall, precision, F1-Score, accuracy, and confusion matrix. These evaluation measures were used to evaluate the model’s performance.

This research compared the suggested DNDF method to the reference approaches of 2D-CNN, Xgboost, and RF (random forest) using the CICIDS 2017 dataset. On the CICIDS 2017 dataset, the accuracy delivered using the Xgboost approach was 99.7%, in contrast to the accuracy delivered by the 2D-CNN strategy, which was 90.6%. There is an opportunity for development in how the Xgboost technique handles intricate patterns and picks up fine features in network traffic data. Although the Xgboost methodology is comparable to the 2D-CNN method, there is room for improvement. When it was applied to the CICIDS 2017 dataset, RF outperformed the previous two algorithms with a level of accuracy that was equal to 99.9 percent. Ensemble learning is utilized to achieve high accuracy in traffic flow-based intrusion detection. It is accomplished by using many sensors.

This study has essential application implications for intrusion detection and network security. With a remarkable precision rate of 99.96%, it ensures improved accuracy in intrusion detection and fewer false alarms, among other noteworthy benefits. The outstanding performance of DNDF strengthens overall defenses by quickly identifying and mitigating malicious network traffic. It can be adapted to network environments found in the real world thanks to its resilience to noisy and unbalanced data. Moreover, the efficiency of DNDF in identifying network traffic instances leads to faster responses to cybersecurity threats. With its ability to be applied to a variety of real-world networks, this innovation points to a bright future for advanced intrusion detection systems and is a valuable tool for boosting cybersecurity across sectors and organizations.

The experimentation was extended to include the CICIDS2018 dataset for evaluating the model’s performance. It was found that the model performed exceptionally well compared to the CICIDS 2017 dataset, achieving 100% accuracy, while the accuracy for the CIS1DS2017 dataset was 0.9993. Additionally, the loss for the 2017 dataset was 0.0027, whereas the loss for the 2018 dataset was 6.2132 × 10^−4^.

This study provides a promising intrusion detection technique but has several notable shortcomings. Its primary reliance on a single dataset raises questions about how well it can be applied to network traffic in the real world. The lack of a thorough discussion of scalability and resource requirements may make it challenging to implement in large-scale networks. The model’s interpretability is poor, and it is still unclear how well it can handle real-time processing. There are few comparisons to cutting-edge solutions, and aspects of security evaluation need more research. In conclusion, operational deployment factors such as integration, scalability, and adaptability demand more focus. While DNDF demonstrates promise, these drawbacks must be resolved to ensure that it can be used effectively in various network security scenarios.

## 7. Conclusions

This study applied the DNDF model to analyze network data, introducing an innovative approach that combines deep neural networks with decision forests to achieve accurate classification and interpretability in network traffic analysis. Our research yielded significant contributions and insights into the DNDF model’s applicability. First, we carried out comprehensive performance assessments utilizing the CICIDS 2017 and CICIDS 2018 datasets and a custom dataset. This was performed to showcase the DNDF model’s exceptional capability in precisely categorizing network data and detecting malicious or irregular activities. Next, we focused on explaining and interpreting the DNDF model by analyzing decision rules and feature importance generated with the decision forest component. This capability is crucial for understanding network traffic patterns and identifying potential threats. Additionally, this study comprehensively investigated the relevance of DNDF model features, significantly improving categorization accuracy, particularly for network assault or anomaly detection. We also conducted a comparative study, highlighting the DNDF model’s advantages over other approaches for analyzing network traffic due to its enhanced performance, durability, and efficiency. Moreover, we explored real-world applications, such as anomaly detection and network security enhancement, emphasizing the DNDF model’s role in proactive network monitoring and response. Addressing scalability and efficiency concerns, we examined the model’s computing requirements, memory usage, and training/testing time, confirming its suitability for real-time and high-throughput network settings.

Our study advances network traffic analysis using the DNDF model, showcasing its superior performance, interpretability, and feature significance analysis. We suggest potential improvements and offer findings that can guide the development of more efficient network security solutions, enhancing proactive threat detection and mitigation.

Future research for the DNDF model in network traffic analysis involves exploring scalability and efficiency in IoT and IIoT networks [47], enhancing robustness against adversarial attacks through defense mechanisms [48], and investigating hardware acceleration methods for real-time implementation in edge devices, ultimately improving network security measures [49]. Additionally, considering detection overhead is crucial for expanding upon this study and further advancing its findings. Precise measurements of detection overhead serve as guiding metrics for creating security solutions that are both more effective and resource-efficient, effectively addressing the ever-evolving challenges within the realm of network security. Furthermore, developing or leveraging real-world network scenarios and specifying intrusion parameters for different data transmissions are crucial steps in expanding the scope of the proposed DNDF approach. These steps will facilitate comprehensive testing and seamless comparisons with other datasets, further enriching the research’s depth and applicability.

## Figures and Tables

**Table 1:** List of related works.

Ref.	Datasets	Methods	Accuracy
[2]	▪ CICIDS 2017 and NSL-KDD dataset.	▪ Deep learning and deep neural network.	▪ Accuracy of 99.6% and 99.4% for NSL-KDD and CICIDS 2017 datasets.
[4]	▪ Data were collected from ISCXVPN2016.	▪ ML, deep learning, reinforcement learning, CNN, KNN, and enhanced deep reinforcement learning (EDRL) algorithm.	▪ The accuracy for the EDRL algorithm is 97%, the mean false positive value is 2.65%, the mean precision is 97.4%, and the mean false negative value is 2.57%.
[7]	▪ USTC-TFC2016, CIC17 and UTSA21 datasets.	▪ Deep learning, CNN, 1D-CNN, 2D-CNN. ▪ ML, R.F., KNN, and SVM.	▪ Accuracy: 90.6%.
[8]	▪ CICIDS 2017 dataset.	▪ SMOTE algorithm, XGBOOST method, and R.F.	▪ Accuracy: 99.7%.
[9]	▪ CICIDS 2017 dataset.	▪ ML, decision tree, RF, and correlation-based feature selection technique.	▪ F1-Score of 0.99; accuracy of RF is 99.86%
[12]	▪ CICIDS 2017 and UNSW-NB15 datasets.	▪ ML, RF, and feed-forward neural networks.	▪ The obtained accuracy for the CICIDS 2017 dataset is 99.8%, and 93.5% for the UNSW-NB15 dataset.
[17]	▪ USTC-TFC2016 and CIC17 Datasets.	▪ ML, transmission control protocol, user datagram protocol, naive Bayes, and decision tree.	▪ Accuracy: 99.5%.
[36]	▪ UNSW-NB15 and CICIDS 2017 datasets.	▪ 1D CNN and random forest model.	▪ Accuracy: 96%.

**Table 2:** CICIDS 2017 data frame.

Port Destination	Flow Duration	Total Fwd Packets	Total Backward Packets	Total Length of Fwd Packets	Total Length of Bwd Packets	Fwd Packet Length Max	Fwd Packet Length Min	Fwd Packet Length Mean	Fwd Packet Length Std
54865	3	2	0	12	0	6	6	6.0	0.0
55054	109	1	1	6	6	6	6	6.0	0.0
55055	52	1	1	6	6	6	6	6.0	0.0
46236	34	1	1	6	6	LO	6	6.0	0.0
54863	3	2	0	12	0	6	LO 6	6.0	0.0

**Table 3:** Dataset properties for traffic analysis of the network.

Basic Flow Features	Source and destination I.P. addresses. Source and destination port numbers. Protocol type (TCP, UDP, etc.). Flow duration.
Traffic Features	Total number of packets. Total number of bytes. Packet and byte rate.
Payload Features	Average, minimum, and maximum packet and byte sizes. The standard deviation of packet and byte sizes.
Network Protocol Features	Number of TCP, UDP, and ICMP packets. Number of HTTP, DNS, and FTP packets.
Statistical Features	Distribution of packet and byte sizes. Statistical measures such as mean, variance, skewness, and kurtosis.
Label (Target Variable)	Indicates the class or category of the network traffic flow (e.g., normal traffic, specific attack types, anomalies).

**Table 4:** CICIDS 2018 data frame.

Protocol	Timestamp	Flow Duration	Fwd Pkts Tot	Bwd Pkts Tot	TotLen Fwd Pkts	TotLen Bwd Pkts	Fwd Pkt Len Max	Fwd Pkt Len Min	…	Fwd Seg Size Min	Active Mean	Active Std	Active Max
0	14/02/2018 08:31:01	112,641,719	3	0	0	0	0	0	…	0	0	0	0
0	14/02/2018 08:33:50	112,641,466	3	0	0	0	0	0	…	0	0	0	0
0	14/02/2018 08:36:39	112,638,623	3	0	0	0	0	0	…	0	0	0	0
6	14/02/2018 08:40:13	6,453,966	15	10	1239	2273	744	0	…	32	0	0	0
LO 6	14/02/2018 08:40:23	8,804,066	14	11	1143	2209	744	0	…	32	0	0	0

**Table 5:** Data frame of the custom network traffic dataset.

Source	Destination	Protocol	Length	Info	Flow Duration	Num_Packets Per_Flow	Flow_Size	Label	MinPacketLength	MaxPacketLength	AvgPacketLength	TotalPackets 1
104.18.37.228	192.168.8.106	15	1094	443 > 64364 Len = 1052	16.07158	1537.0	1,736,392.0	attack	67	1242.0	1129.728042	9443.0
104.18.37.228	192.168.8.106	15	1242	443 > 64364 Len = 1200	16.07158	1537.0	1,736,392.0	attack	67	1242.0	1129.728042	9443.0
104.18.37.228	192.168.8.106	15	1095	443 > 64364 Len = 1053	16.07158	1537.0	1,736,392.0	attack	67	1242.0	1129.728042	9443.0
104.18.37.228	192.168.8.106	15	1242	443 > 64364 Len = 1200	16.07158	1537.0	1,736,392.0	attack	67	1242.0	1129.728042	9443.0
104.18.37.228	192.168.8.106	15	1098	443 > 64364 Len = 1056	16.07158	1537.0	1,736,392.0	attack	67	1242.0	1129.728042	9443.0

**Table 6:** Null entries in a data frame.

Destination Port	0
Flow Duration	0
Total FWD Packets	0
Total Backward Packets	0
Total Length of FWD Packets	0
	………..
Idle Mean	1
Idle Std.	1
Idle Max	1
Idle Min	1
Label	1

**Table 7:** Updated data frame after removing null entries.

Destination Port	0
Flow Duration	0
Total FWD Packets	0
Total Backward Packets	0
Total Length of FWD Packets	0
	………..
Idle Mean	0
Idle Std.	0
Idle Max	0
Idle Min	0
Label	0

**Table 8:** Thirty-nine selected correlated features.

PSH Flag Count	Flow Packets/s	Fwd Packet Length Max	Init_Win_bytes_forward	Fwd Avg Bulk Rate	Bwd Avg Packets/Bulk	URG Flag Count	Destination Port	Bwd IAT Std
0.0	666666.66670	6	33.0	0.0	0.0	0.0	54865	0.0
0.0	18348.62385	6	29.0	0.0	0.0	1.0	55054	0.0
0.0	38461.53846	0	29.0	0.0	0.0	1.0	55055	0.0
0.0	58823.52941	6	31.0	0.0	0.0	1.0	46236	0.0
0.0	666666.66670	6	32.0	0.0	0.0	0.0	54863	0.0

**Table 9:** Categorical target variable.

0	Benign
1	Benign
2	Benign
3	Benign
4	Benign
	……..
525468	Benign
525461	Benign
525462	Benign
525463	Benign
525464	Benign

**Table 10:** Standardized features data frame.

Array ([[0.	−0.62552915	−0.25022434,	0.00840025,
U	−0.16757888]		
[0.0.	−0.62080808, −0.16757888]	−0.24989545,	−0.0092991,
[0. I	0.00989455, −0.16757888],	−0.2501323	−0.00536591,
[0.	0.00989455, −0.16757888],	−0.25018099	−0.00536591,
[0 . 0.	−0.62512906, −0.11300009],	−0.25022265	−0.00339931,
[0 . 0.	−0.56367516, −0.14938595]]	0.00143074	−0.0092991

**Table 11:** Evaluation metrics of the proposed model.

Evaluation Metric	Performance Value
Mean accuracy	0.9999
Test accuracy	0.9995
Macro precision	0.9997
Macro recall	0.9831
Macro F1 score	0.9912

**Table 12:** Performance evaluation metrics values.

Evaluation Metric	Performance Value
Mean accuracy	0.9968
Test accuracy	0.9968
Macro precision	0.9962
Macro recall	0.9967
Macro F1 score	0.9964

**Table 13:** Performance evaluation values on the testing subset of the CICIDS 2018 dataset.

Evaluation Metric	Performance Value
Mean accuracy	0.99995
Test accuracy	0.99995
Macro precision	0.99992
Macro recall	0.999939
Macro F1 score	0.999930

**Table 14:** Comparison between different methods.

Reference	Approach	Accuracy	Dataset
[7]	2D-CNN	90.6%	CICIDS 2017
[8]	XGBOOST	99.7%	CICIDS 2017
[9]	RF	99.8%	CICIDS 2017
**Our Approach**	DNDF	99.96	CICIDS 2017

## Data Availability

The dataset is available on reasonable request.
